# Two contemporaneous morphs of fossil *Chanos* Lacepède, 1803 (Gonorynchiformes, Chanidae) from Paleocene (Danian) outcrops near Palenque (Mexico) revealed by geometric morphometrics indicate conservatism in milkfishes after the K/Pg boundary

**DOI:** 10.1371/journal.pone.0313912

**Published:** 2025-03-05

**Authors:** Alberto Guadarrama, Kleyton M Cantalice

**Affiliations:** 1 Posgrado en Ciencias Biológicas, Universidad Nacional Autónoma de México, Ciudad de México, México; 2 Departamento de Paleontología, Instituto de Geología, Universidad Nacional Autónoma de México, Ciudad de México, México; Università degli Studi di Torino, ITALY

## Abstract

We described chanid material from the Paleocene (Danian) localities of División del Norte and Belisario Domínguez near the archeological site of Palenque, Chiapas State, southeastern Mexico. The parsimony-based morphological phylogeny indicates that the specimens are closely related to the extant milkfish, *Chanos chanos* (Teleostei, Ostariophysi), and the comparative anatomy reveals a remarkable qualitative similarity of almost every visible bone. Among the synapomorphies for the genus *Chanos*, is a pleurostyle or caudal complex, which is missing in all other chanid fossils. Extant milkfish are highly variable in meristic and morphometric traits, and we found a signal for quantitative variation with geometric morphometrics tools in the fossil sample. We first dealt with post-mortem body torsion and missing landmarks. The main analysis shows a pattern of two forms present in both localities. A group of specimens shows a bigger head and deeper body than the slender and smaller head of the rest, implying that two types of milkfish coexisted in time and space. We tested for allometry and explored scenarios that can explain the patterns, such as sexual dimorphism or two sympatric and closely related species for the morphotypes, and differential resource utilization for the jaw, head, and depth variations. Furthermore, we argue that, alongside the morphological stasis, *Chanos* has conserved the life history trait of fry migration towards near-shore nurseries through protracted time (~63mybp). We infer the fish were juvenile, and the paleontological assemblage and taphonomy suggest that the localities exhibit the influence of both marine and transitional environments.

## Introduction

Gonorynchiformes is an order of teleostean fishes composed of three families: Chanidae, Gonorynchidae, and Kneriidae. This clade is the sister group to the remaining ostariophysians, the fishes with functioning Weberian Apparatus gathered into Otophysi: Cypriniformes, Gymnotiformes, Characiformes, Siluriformes, and Cithariniformes [1]. In contrast to otophysians, a group that includes over a quarter of extant fish diversity, fossil and extant diversity of Gonorynchiformes is depauperate [1–3]. They probably have a Pangean or Tethyan origin and appear in the fossil record during the Berriasian (Lower Cretaceous) period [4].

The milkfish *Chanos chanos* (Fabricius, 1755) is the sole extant Chanidae. It is a large, long-lived, pelagic, generalist, euryhaline, plastic, and pantropical Indo-Pacific fish [5,6]. *Chanos chanos* can show statistically significant morphometric and meristic variability between distant allopatric populations, just as between close panmictic populations [7–10]. In the nineteenth century, multiple junior synonyms of this species were coined based on geographical variations [6].

The fossil record of Chanidae comprises around 13 genera and 16 species distributed in South America, Africa, Asia, and Europe, spanning from the Lower Cretaceous (Berriasian) to the Miocene (Burdigalian) [2,11–16]. The morphological features of all the chanid genera are roughly similar [14]. Recently, detailed anatomical studies resulted in newly recognized taxa [12–14], but the validity of several fossil species remains questionable [2]. Several named morphs, such as †*Dastilbe crandalli* Jordan, 1910, †*D. batai* Gayet, 1989, and †*D. elongatus* Silva-Santos, 1947, which differ in morphometric proportions [2,17–21]; †*Chanos brevis* (Haeckel, 1854), †*C. zignoi* Kner and Steindachner, 1863, and †*C. forcipatus* Kner and Steindachner, 1863 which differ in vertebrae count, are considered junior synonyms of their type, and their differences are generally explained by geographical variation [2,17]. The species †*Rubiesicthys gregalis* Wenz, 1984 has two coexisting morphs, inferred to be a case of sexual dimorphism [22].

Here, we present newly described chanid fossil forms, retrieved from two small and shallow paleontological sites 9.5 km south and 6 km southeast respectively to Palenque, Chiapas State, southeastern Mexico. These sites are the Belisario Domínguez (BD) and the División del Norte (DN) quarries, previously reported by different authors e.g., [23,24] as Danian ( ≈ 63 mybp) outcrops of the Paleocene Tenejapa-Lacandón geological unit. These two formations are contemporaneous and laterally continuous, deposited in the periphery of a shallow carbonated platform [25]. Both quarries show parallel and finely laminated carbonated strata of yellow-cream-colored marls in an 8 to 15 cm sequence. Little is known about other geological aspects of these localities, and no stratigraphic column exists.

The paleontological structure is similar in both localities, consisting primarily of plant and fish fossils. Currently, the fish assemblage of these sites includes Pycnodontiformes, Anguilliformes, Clupeiformes, and different acanthomorph clades e.g., [23,26]. The quality of preservation and geographical-temporal distribution of these fossils near the Chicxulub impact crater and the K/Pg boundary associated with the mass extinction event highlights the importance of these localities in the understanding of the origin and tempo of the modern marine fish fauna [23,27].

## Materials and methods

The specimens selected to perform this work are the best-preserved, most-complete, and least-deformed for a total of 54 specimens housed in the *Colección Nacional de Paleontología* (CNP), *Instituto de Geología*, *Universidad Nacional Autónoma de México*, at Mexico City, Mexico. (see Supporting information, S1 PDF). No permits were required for the described study, which complied with all relevant regulations. Most of these fossils required little preparation, but we removed rock sediments from some of the fossils with fine brushes, needles, and air scribes under a stereoscopic microscope.

Additionally, we made rubber molds of the holotype and selected specimens. One specimen of extant *C. chanos* from the CNP, CMR 1259, was cleared and stained following Esguícero and Bockmann [28] protocol for the comparative anatomy analysis. The nomenclature of osteological elements follows Grande and Poyato-Ariza [29], Grande and Arratia [30], Poyato-Ariza et al. [31], and Koch and Moritz [32].

### Phylogenetic analysis

We were first interested in the relationships within Chanidae of the examined material, testing the original hypothesis of a chanid and gonorynchiform fish. For this, we performed a parsimony-based phylogenetic analysis based on the morphological matrix of Ribeiro et al. [13], which works upon the original in Poyato-Ariza et al. [31] and the next iteration of Amaral and Brito [11]. Being a revision of chanid relationships, it excludes myologic characters and most of the Kneriidae and Gonorynchidae taxa.

We added a single composite coding of †*Chanos chautus* sp. nov*.* in Mesquite version 3.81 [33], as the individual coding of morphotypes would be identical. We modified the state 0 to 1 of character 75 (abutting contact of anterior neural arches) for *C. chanos* per our observations of the recent material. Since their predorsal neural arches are autogenous and large, especially the first five, they are in contact with those adjacent anteriorly and posteriorly without overlapping.

Parsimony analysis and character mapping were performed in TNT v.1.6 [34], indicating †*Diplomystus* Cope, 1877 as the outgroup as explained in the original work [31]. Characters were unweighted and treated as unordered. The maximum memory for trees was increased to 10,000. Per the small number of terminal taxa (15), we analyzed the dataset by the exact tree search implicit enumeration (or branch and bound), which finds all optimal topologies. The clade consistency was obtained by bootstrap analysis with 1,000 pseudoreplications. The discrete matrix is available in Supporting information (S1 file).

### Geometric morphometrics

#### Data acquisition and curation.

Only laterally preserved and complete or semi-complete specimens (44) from the original comparative anatomy sample were selected for geometric morphometrics (GM). We allowed a maximum of four lost landmarks, whether completely absent or preservationally distorted; eight specimens had one missing landmark (mlm), one had two mlm, three had three mlm, and one had four mlm (see Supporting information, S1 PDF). Fifteen specimens were not bent, but we allowed specimens with different degrees of dorsoventral torsion. To keep spurious ontogenetic effects to a minimum we also restricted our dataset to fish with a standard length (SL) > 35 mm.

Standardized high-resolution photos (e.g., with the same tripod, same light, and same settings) were taken with a Nikon DSLR D5500 camera and an AF-S Micro NIKKOR 60 mm lens to prevent any deformation of images and perspective. Images of specimens preserved on their right side were mirror-reflected to standardize all specimens on their left side. Images in.jpg format were converted to.tps format with tpsUtil [35].

Fifteen landmarks, 14 homologous (biological/topological correspondence), and one reference point (14) [36,37] were digitized in tpsDig2 [38]: (1) snout tip, (2) posterior end of frontals, (3) origin of the dorsal fin, (4) insertion (end) of the dorsal fin, (5) anteriormost dorsal procurrent ray of the caudal fin, (6) end of the vertebral column (pleurostyle and hypural plate 1), (7) anteriormost ventral procurrent ray of the caudal fin, (8) insertion of the anal fin, (9) origin of the anal-fin, (10) origin of the pelvic-fin rays, (11) origin of the pectoral-fin rays, (12) cleithrum-supracleithrum articulation (posteriormost edge of the opercle), (13) interopercle-subopercle articulation (anteroventralmost edge of opercle), (14) angle between the arms of the preopercle’s ridge, and (15) ascending process of parasphenoid (posteroventral margin of the orbit).

A table describing the type, position, definition, and further notes on precise landmark placement is available in Supporting information (S1 PDF). This skeletal anatomy landmark system uses other 2D ostariophysan fish landmark systems as reference e.g., [39]. During design, we accounted for the general preservation of the specimens while sufficiently characterizing the shape and its variation.

The lost landmarks of 15 specimens of the raw dataset (S2 File) were estimated with the *estimate.missing()* function of *geomorph* [40,41] in RStudio Build 554 [42]. Variations of tpsDig2 and tpsUtil [35,38,43] unbending procedure were performed to correct dorsoventral torsion. This protocol consists of adding extra landmarks on a midline, in this case, 8 or 10 landmarks, whether considering only postcranial torsion or along all of SL, respectively) along the spine; tps then calculates the perpendicular deviations on a straight line according to a cubic or quadratic curve fit. Finally, landmarks 16 to 25 were removed for further analysis. Four specimens had particularly low (<0.25) regression scores and did not adjust to any unbending variation, and thus were not used in the main analyses, leaving a final *n* of 40 for GM in the not-bent +  unbent sample (S5 File). Selected landmark-based linear morphometrics (interlandmark distance) measurements were calculated using the measurement tool of tpsDig2 [37] (see Supporting information, S1 PDF).

Bent or incomplete specimens are sometimes removed from the datasets [e.g., 38] as they introduce spurious variation. Nonetheless, different unbending protocols have yielded success in minimizing the effect of torsion [44–46]; among these, tps-unbending was found to be the best option for dealing with arching in fossils, especially for multivariate analyses, as assessed in the Chanidae of Las Hoyas (Cretaceous, Spain) [47]. As in this study, they also found that most specimens adjusted better to a cubic rather than a quadratic fit, probably as arching in fossils is often irregularly sigmoidal [47]. Also, Arbour and Brown [48] demonstrated with different manipulations of empirical datasets that estimation can be preferred to landmark or specimen deletion in the majority of cases, particularly in datasets with low disparity and if using the regression method (implemented in *estimate.missing()* function) as their estimates fell within the digitization error range.

#### Cluster analyses.

All further GM analyses were performed in RStudio Build 554 [42] with the R package *geomorph* v.4.0 [40,41]. The concerns regarding replicability derived from preservation and ambiguity of landmark definition were addressed by testing digitization error [49] in the estimated sample. This was calculated by performing a non-parametric and distance-based Procrustes analysis of variance (ANOVA), comparing digitization replicates of the total sample (bent and not-bent) taken on separate days (one month apart, different researcher, AG, and KMC) through the *procD.lm()* function and 999 iterations and default settings, using as linear model: Procrustes coordinates (shape) explained by the independent variables days and individuals. The function uses Procrustes distances among specimens as a measure of SS instead of covariance matrices, preferable for small sample sizes. It evaluates such values through permutation and a test-statistic analog to Fishers’s *F*-ratio [50,51].

For the main analyses, position, orientation, and scale effects were removed using the generalized Procrustes superimposition (GPA) method using the *gpagen()* function, producing only shape variables [46,52]. Using the *plotOutliers()* function, we searched for outliers, which plots specimens according to their Procrustes distance from the mean shape. A principal component analysis (PCA) of Procrustes coordinates was performed to identify the major patterns of shape variation [53–55] using the *gm.prcomp()* function in the not bent (n = 15), ‘total’ (n = 44, not-bent and bent), and ‘unbent’ (n = 40, not-bent and unbent) datasets (S2-5 Files). We visualized the morphospace and described shape changes for the first three PCs (differences between minimum and maximum) through the *plotRefToTarget()* function. Up to this point, no a priori groups were considered, as our null hypothesis was a single variable shape. Since visualization signaled two unambiguous groups (no-overlapping) on the unbent dataset (see Results), 23 specimens of henceforth morphotype 1 (M1) and 17 of morphotype 2 (M2), we decided to test the significance of differences between the two and the effect of size, despite the small sample sizes. We, therefore, performed a Procrustes ANOVA through the *procD.lm()* function with 99 (exploratory run, per the small sample) and with 999 permutations and default settings, the linear model being: shape explained by morphotypes and log-transformed centroid size (CS) (i.e., does shape change with size?) and the interaction between the two kinds of independent variables (i.e., do morphotypes differ in size?). CS is traditionally used as a proxy of size in geometric morphometrics e.g., [56–58], it has no relation to shape and is defined as the square root of the sum of the squared distances of all landmarks to their centroid [36,59]. We visualized the allometric relationship of shape and size according to our ANOVA fit via *plotAllometry()* (PredLine method, a regression) with CS as the size predictor for the fitted PC shape values.

As we opted for a Procrustes permutational ANOVA because of our sample size, we used the whole shape instead of using only the principal axes of variation (variance-covariance matrices), whether PCs or relative warps (RWs) [e.g., 45]. We also opted for PCA and not canonical variates analysis (CVA) e.g., [60,61], as it requires a priori grouping while maximizing differences between these groups [62,63]. We did not add locality as a variable as ¾  of specimens come from BD and 10 (seven M1, three M2) from DN (See Supporting information, S1 PDF). Geometric morphometrics datasets and the R code are available in Supporting information (S2-S6 Files).

### Nomenclatural Acts

The electronic edition of this article conforms to the requirements of the amended International Code of Zoological Nomenclature, and hence the new name contained herein is available under that Code from the electronic edition of this article. This published work and the nomenclatural acts it contains have been registered in ZooBank, the online registration system for the ICZN. The ZooBank LSIDs (Life Science Identifiers) can be resolved and the associated information viewed through any standard web browser by appending the LSID to the prefix ““http://zoobank.org/”“. The LSID for this publication is: urn:lsid:zoobank.org:pub: 95765763-6EA6-4C00-9094-3F767306C392. The electronic edition of this work was published in a journal with an ISSN, and has been archived and is available from the following digital repositories: PubMed Central and LOCKSS.

## Results

### Systematic Paleontology

**Fig 1:**
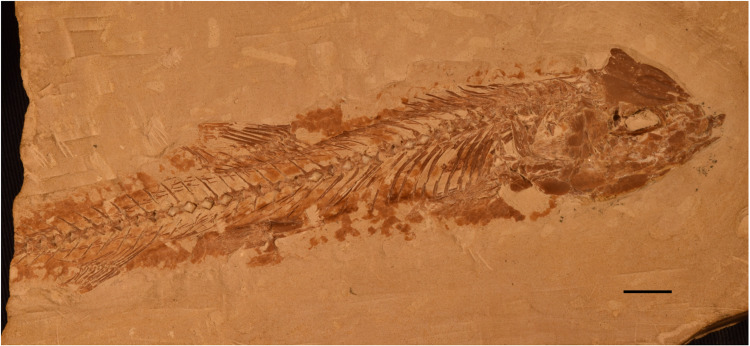
†*Chanos chautus* sp. nov. holotype, IGM 13970.

Division TELEOSTEI Müller, 1845
Infracohort OSTARIOPHYSI Sagemehl, 1885
Series ANOTOPHYSI Rosen and Greenwood, 1970
Order GONORYNCHIFORMES Regan, 1909
Family CHANIDAE Jordan, 1887
Subfamily CHANINAE *sensu* Poyato-Ariza et al., 2010
Tribe CHANINI *sensu* Poyato-Ariza et al., 2010
Genus *CHANOS* Lacepède, 1803
†*Chanos chautus* sp. nov. (Fig 1)

**LSID:** urn:lsid:zoobank.org:act:EB151209-9E8C-4F0D-A444-B529ECE16CC3

**Diagnosis.** †*Chanos chautus* sp. nov. differs from extant *C. chanos* by a combination of features, including the lack of the anterior notch in the mesethmoid, with longer posterior wings arranged closer to each other, and its lateral wings less developed; a shorter predorsal length of 56.14-63.49% of SL, and a smaller number of vertebrae, 35 to 42.

**Derivation of the name.** In honor of the precolonial Mayan city of Palenque, the composite specific epithet derives from two words of the Mayan language: *ka’* meaning two, and *utus* meaning face. Therefore, referencing the morphotypes and their head variation, the complete name means ‘the two-faced *Chanos*’.

**Holotype.** IGM 13970, 147.87 mm of SL. A M1 specimen preserved on its right side with most of the skull bones preserved but the tail is missing.

**Paratypes.** IGM 13978, a complete M2 specimen preserved on its right side, 115.22 mm of SL. IGM 13977, a M1 specimen without anal fin, 55.03 mm of SL, and IGM 13984 a complete M2 specimen with a detailed caudal skeleton, 53.99 mm of SL; both preserved near one another in the same slab.

**Referred material**. 50 specimens, recorded as IGM13971-IGM14013 (see S1 PDF for a list with locality and SL of all material).

**Type locality and horizon.** Danian (63mybp, Paleocene) strata of Tenejapa or Lacandón formations. Belisario Domínguez (17° 25’ 28.60“ N y 91° 58’ 46.80” O) and División del Norte (17° 16’ 12.17” N y 97° 40’ 40.7” O) quarries, Chiapas State, southeastern Mexico.

### Description

#### General body features.

Standard length ranges from 35.8 to 147 mm, selected landmark-based linear morphometrics are shown in Table 1. The body shape is fusiform. The head is triangular, with a small terminal mouth, and large eyes. The head length and depth occupy 26.58-43.58% and 14.65-24.42% of the SL, respectively. The maximum body depth is near the dorsal fin and represents 20.8-22.1% of the SL. The dorsal fin is in the middle of the body, originating at 56.14-63.49% of the SL, whereas the anal fin is short and located far in the back of the trunk, its origin being at 76.68-90.68% of the SL. The paired fins are triangular and lie near the ventral edge of the abdomen. The pelvic fin is abdominal, positioned opposite to the middle part of the dorsal fin, and barely exceeds its posterior end. The caudal peduncle is shallow. The caudal fin is homocercal, deeply forked, and exceeds the maximum body height.

**Table 1:** Landmark-based linear morphometrics measurements.

	Head length SL% [1–5]	Head depth SL% [2–13]	Body depth SL% [3–10]	Predorsal length SL% [1–3]	Preanal length SL% [1–9]	Caudal peduncle depth SL% [5–7]
†*Chanos chautus* sp. nov. n = 40	26.58-43.58 (33.65)	14.79-24.42 (19.26)	13.9-26.79 (20.76)	56.14-63.49 (58.61)	76.68-90.68 (83.06)	5.26-12.42 (8.72)
†*Chanos chautus* sp. nov. M1 (n = 23)	26.73-31.75 (29.03)	14.65-18.8 (16.87)	13.9-20.95 (18.02)	56.14-61.53 (57.94)	79.91-86.52 (82.70)	5.26-10.12 (8.11)
†*Chanos chautus* sp. nov. M2 (n = 17)	35.62-43.58 (39.90)	20.32-24.46 (22.5)	22.09-26.79 (24.46)	57.3-63.49 (59.5)	79.59-90.68 (83.55)	7.83-12.42 (9.55)

Measurements were taken as the inter-landmark distance shown in square brackets; the mean of measurements are shown in brackets.

#### Neurocranium.

**Fig 2:**
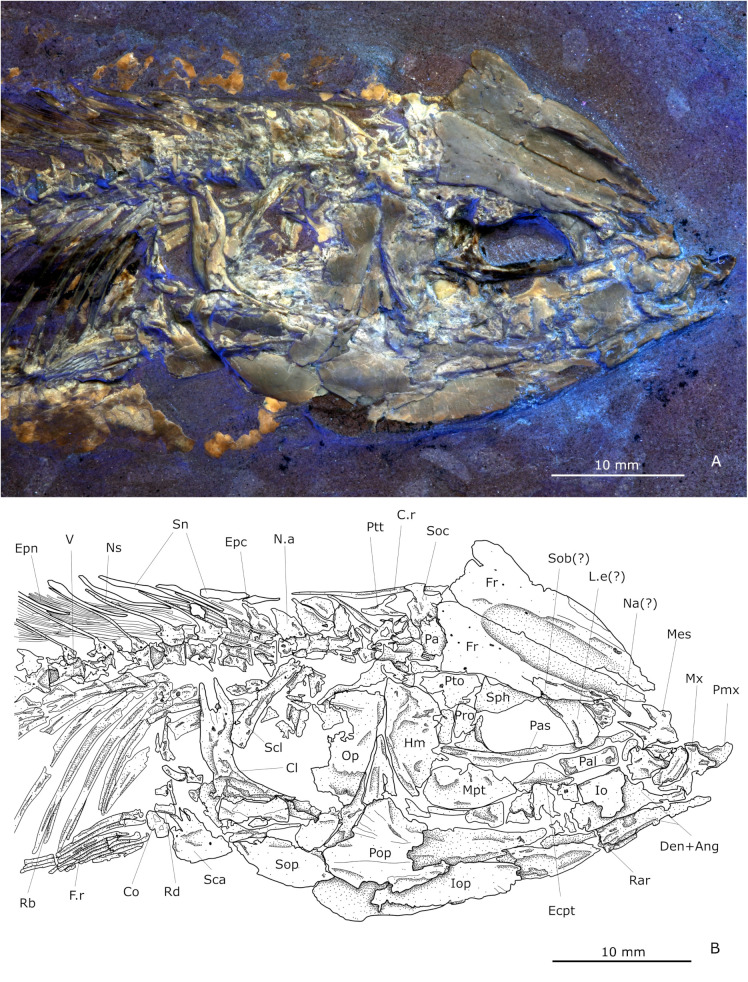
Head of the holotype IGM 13970. **(A)** Photography under UV light. **(B)** Schematic drawing. Abbreviations: Ang, anguloarticular; Cl, cleithrum; Co, coracoid; C.r, Cranial rib; Den, dentary; Entp, entopterygoid, Epc, epicentrals, Epn, epineurals; Fr, frontals; F.r, Fin rays; Hm, hyomandibula; Io, infraorbital; Iop, interopercle; L.e, lateral ethmoid; Mes, mesethmoid; Mpt, metapterygoid; Mx, maxilla; Na, nasal; N.a., neural arch; Ns, neural spines; Op, opercular; Pa, parietal; Pal, autopalatine; Pas, parasphenoid; Pop, preopercular; Pro, prootic; Pto, pterosphenoid; Ptt, postemporal; Rar, retroarticular; Rb, rib; Rd, radial; Sca, scapula; Scl, supracleithrum; Sob, supraorbital; Soc, supraoccipital; Sop, subopercle; Sph, sphenotic; V, vertebrae centrum.

In dorsal view, the skull roof is triangular, about two times wider than long, and rostrally tapered (Fig 2). In its dorsal view, the mesethmoid is a complex star-like shaped bone, with a rounded anterior end, a pair of wings or lateral processes, and a posterior pair of long stout processes overlapped by the anterior end of both frontal bones. Lateral ethmoids can only be identified by their position and part of their wing protruding posterior to the mesethmoid, separating the nasal capsule from the orbit.

The frontals are the largest bones in the skull and form the majority of the cranium in dorsal view; they exhibit a straight interfrontal suture, and together form an arrowhead-shaped cranial surface with a deep and broad interfrontal depression extending from their ethmoidal end up to the posterior part of the orbit (Fig 2B). Each frontal lateral edge exhibits a medial constriction behind the orbit and above the sphenotic bone. The posterior edge includes a small and rounded medial notch in contact with the supraoccipital bone.

Most of the bones in the postorbital region of the skull are not well preserved. The skull shows the latero-parietal condition, in which parietals are separated by the supraoccipital bone. Parietals are reduced, flat, smooth, and square-like bones. In the dorsal view, the supraoccipital is a complex hexagonal or star-like bone that sutures the frontals anteriorly, the exoccipitals posteroventrally, and the parietals laterally. The supraoccipital has an elongated and bifid posterior crest with two sets of 10-12 brush-like filaments distally.

The sphenotic is a small, somewhat triangular bone that borders the dorsoposterior part of the orbit and sutures the anterior pterotic edge. Lateroventrally articulated to the respective frontal and parietal, the pterotics are elongated and complex bones. They exhibit a foramen and have distal main branch-like projections posteriorly. The exoccipitals form a flat arrowhead-like structure pointing posteriorly, overlapping the basioccipital. The parasphenoid is a slender, straight, toothless bar-like structure that extends through the lower part of the orbit and reaches the ethmoid part of the skull. It has an ascending process at the posterior edge of the orbit. There are large cephalic ribs in the posterodorsal region of the skull, but its articulation is ambiguous. Other elements such as vomer, intercalary, extrascapular, prootic, and epiotic cannot be described unambiguously, all seem to be present but hidden or fragmented.

#### Circumorbital series.

Overall, the bones of the circumorbital series are poorly preserved. However, fragments of an indeterminate number of infraorbitals cover the dorsal region of the cheek and part of the postorbital region of the skull. A single thick supraorbital forms the anterodorsal border of the orbit (Fig 2B). A small and flat lacrimal bone probably covers the ethmoid region of this fish.

#### Jaws.

**Fig 3:**
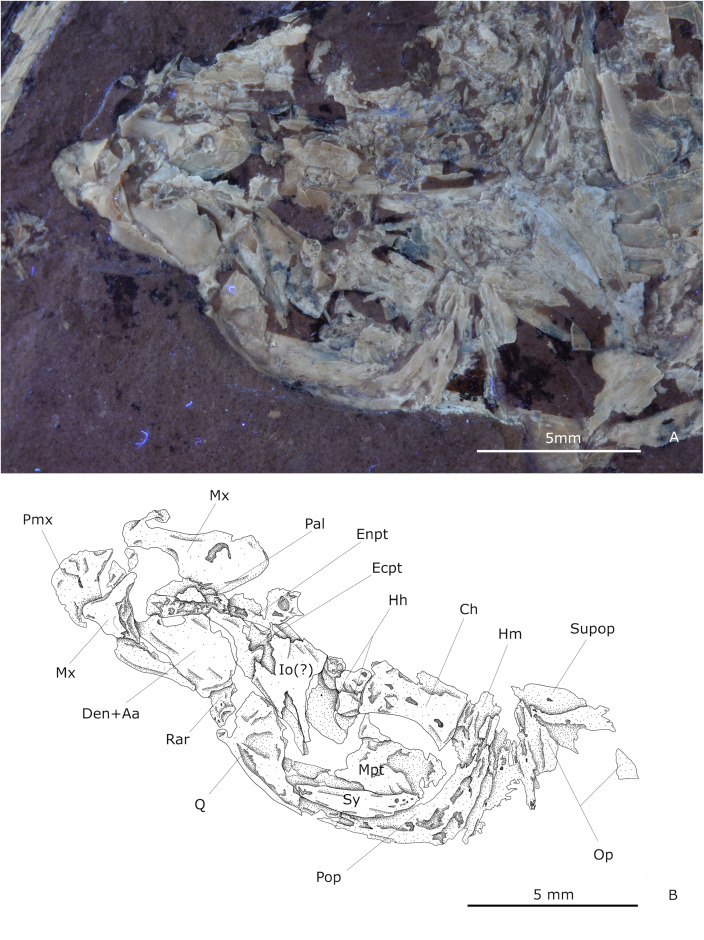
Jaws and suspensorium of IGM 13974. **(A)** Photography under UV light. **(B)** Schematic drawing. Abbreviations: Aa, anguloarticular; Ch, ceratohyal; Den, dentary; Ecpt, ectopterygoid; Enpt, endopterygoid; Hh, hypohials; Hm, hyomadibula; Io, infraorbital; Mpt, metapterygoid; Op, opercular; Pal, autopalatine; Pmx, premaxilla; Q, quadrate; Rar, retroarticular; Supop, suprapreopercle; Sy, symplectic.

As in all gonorynchiforms, the jaw lacks teeth. The premaxilla is a concave-convex half-moon-like bone without an ascending process. It has a nearly straight ventral edge and a spiny-like posteroventral process that partly envelops the maxilla (Fig 3B). The majority of M2 specimens seem to have a marginally protruding snout, perhaps by a slight anterior displacement of the premaxilla. The maxilla is a complex bone with a dorsoventral constriction separating it into two parts. The anterior end is T-shaped, with a very shallow maxillary facet and process for the articulation with autopalatine just before the ventral and dorsal anterior spiny processes. In contrast, the posterior part forms a broad and oblong posterior expansion. In M2 the maxilla appears to be bate-shaped, slightly straighter and more elongated than the more curved and compact posterior section of M1. Furthermore, M1 possesses the maxillary facet and process for articulation with the autopalatine not visible in any of M2 specimens. However, only fragmented maxillae were found.

The general shape of the lower jaw is triangular and consists of the dentary and anguloarticular-retroarticular bones (Fig 3B). The dentary has a shallow symphysis and a high and rounded coronoid process occupying the posterior two-thirds of this bone. As in most Chanidae, the coronoid process has a notch in its anterior ascending border (Fig 3B). The alveolar part of the dentary is thick, low, and tapered anteriorly. In lingual view, the anguloarticular shows a tightly U-shaped suture with the dentary and posteriorly forms an articular process in which a distal small facet for the quadrate protrudes.

#### Suspensorium.

The autopalatine is an elongated and thick bone with a shallow medial constriction and a relatively broad anterior end. The endopterygoid is large and has a rectangular shape that extends below the orbit. The ectopterygoid is a slender curved bone located between the ventral part of the autopalatine, and the anteroventral edge of the endopterygoid (Fig 3B). These three elements always appear fused, at least by cartilage. The metapterygoid is located posteroventral to the endopterygoid and has a somewhat rectangular shape. (Fig 3B). The quadrate-lower jaw joint is slightly anterior to the orbit. The quadrate is somewhat triangular and has a spherical, conspicuous articular head forming its anterior tip. The anterior border is straight, with a sinuous dorsal edge, and a long stake-like posterior process (Fig 3B). The symplectic is a fusiform bone as long as the quadrate. It articulates ventrally with the quadrate, dorsally with the metapterygoid, and posteriorly with the subopercle (Fig 3B).

The hyomandibula has an axe-like shape with a sharp anteroventral process extended below the orbit. The dorsal border is nearly straight but vaguely inclined anteroventrally articulating with the sphenotic and the pterotic; the anterior surface is curved. In the inner view, the opercular process of this bone is located in the dorsal quarter of its posterior border, from which an articular head projects lightly.

#### Opercular series.

The opercular series is complete and includes mostly thin, unornamented bones. The opercle is kidney-shaped, slightly higher than wide, and has a straight yet inclined anterior edge and a curved rear. In its inner view, the hyomandibular facet occupies the upper quarter of its anterior edge. The opercle overlaps the dorsal part of the subopercle, which is slice-shaped, dorsally straight, ventrally curved, and has a small spiny anterodorsal process.

The suprapreopercle is almost always lost, but some fragments reveal its presence in the anterodorsal border of the opercle. The preopercle is an inverted L-shaped bone, in which the limbs are roughly equal in length and form a 90° anterior aperture angle. It has a conspicuous median ridge and a canal through most of both limbs. The ventral region of this bone covers most of the interopercle: an elongated triangular bone anteriorly tapered, with ventral and dorsal edges nearly straight, and a slightly curved rear.

#### Hyoid and branchiostegal regions.

Most bones in this region are obscured. The hypohials are two conical and similarly sized bones (Fig 3B). The anterior ceratohyal is a rectangular and unpierced bone attached to both hypohials anteriorly and at least four branchiostegal rays ventrally. The posterior ceratohyal is unknown. The urohyal is triangular, elongated, and anteriorly tapered, it exhibits an anterior constriction that separates its small anterior articular head.

#### Vertebral column and intramuscular bones.

The vertebral column consists of 35 to 42 vertebrae including the terminal complex. Among these, 16-20 are abdominal and 19-22 are caudal. The counts include the three anteriormost abdominal centra which often the opercle covers. The centra are well-ossified, cylindrically shaped, and slightly constricted in the middle. These have deep conical intervertebral surfaces and a couple of parallel lateral perforations separated by a longitudinal crest. The anterior five centra are slightly shorter than higher and their neural arches and respective spines are bigger than the rest, they abut without overlap (Fig 2B).

All and only predorsal neural arches are autogenous, with inconspicuous anterodorsal processes and bifid neural spines. Both neural and haemal spines are thin, nearly straight, and tilted posteriorly, forming angles between 45 and 60° on the longitudinal axis of the vertebral column. The abdominal cavity is enclosed by 13 to 14 pairs of pleural ribs. In lateral view, these are curved and have small articular heads. The three anterior ribs are hook-shaped with a deep apicobasal groove, broad bases, and slender tips, while the subsequent ones are rather uniformly slender. The first two centra bear no ribs. About half of the ribs appear to articulate directly on the lateroventral surface of the respective centrum, while the posterior ribs attach with paraphophyses in the posterior half of the centra.

A series of 10 to 13 supraneural bones occupy the interneural spaces of the predorsal region. The first four supraneurals are comparatively thicker and broader, and their dorsal halves bend backward, while the rest are rod-like.

Intermuscular bones include epineural, epipleural, and epicentral bones. There are two sets of epineurals. The predorsal epineurals are more complex and situated closer to the neural arch. Four to five main arms extend from a central body, one posterodorsally and three or four anteroventrally; all arms distally have brush-like ends. Postdorsal and preanal epineurals are Y-shaped, anteriorly bifid, have no brush-like distal ends, and are fixed at the distal third of the respective neural spine. The epineurals placed behind the anal fin rest again closer to the vertebral centra.

The epipleurals are elongated bones associated with haemal arches and spines of caudal centra between the pelvic girdle and the preural 5 or 6. The position and shape of these bones are symmetrically opposed to that of the epineurals placed above. These bones are Y-shaped and lie nearly horizontally below two or three centra. The proximal end is deeply bifurcated. In the middle abdominal region, these bones attach to the distal section of haemal spines. Subsequently, those epipleurals placed behind the anal fin tend to be fixed closer to the haemal arches.

Epicentrals appear to be associated exclusively with abdominal vertebrae and are very small, especially compared to epineurals or epipleurals. They have a Y figure, with two small anterior branches and a long spiny posterior one; it is unclear exactly where they attach to the centra.

#### Pectoral girdle and fin.

The supracleithrum is long, as wide as the first pleural rib, and somewhat curved. It articulates dorsally to the cleithrum, overlapping it. The cleithrum is a heavily ossified and curved bone; with ridges and arms from where more laminar bone extends, mainly dorsally and anteroventrally (Fig 2B).

The coracoid is big, almost as long as the subopercle. It has an axe-like shape, an elongated narrow anterior half articulating medially with the cleithrum, and a deep posterior half articulating with the scapula. The scapular foramen is positioned in the ventral portion of the bone. Four prominent proximal radials and 13 to 16 unsegmented rays are present.

#### Pelvic girdle and fin.

**Fig 4:**
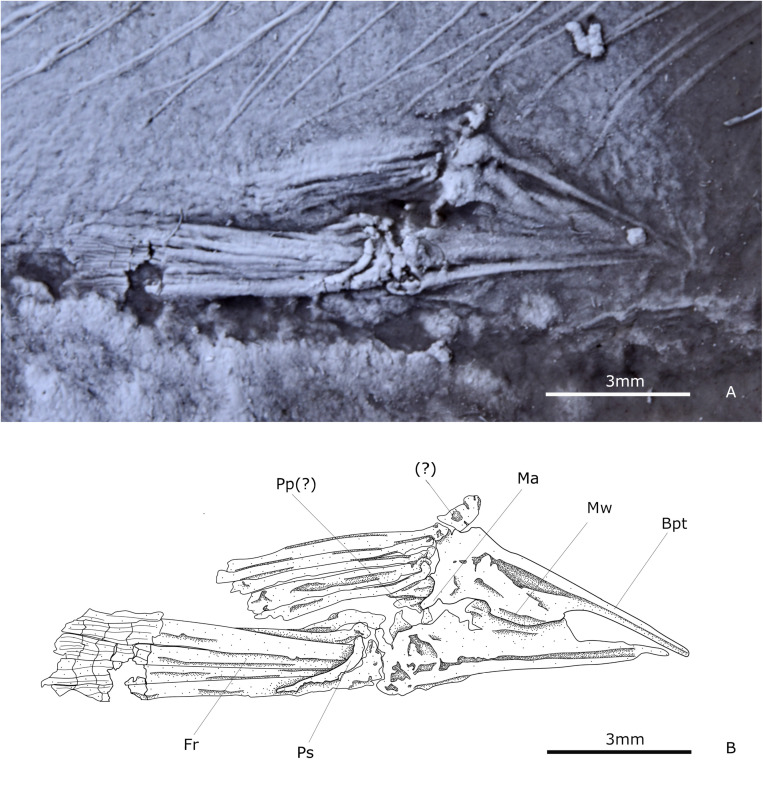
The pelvic fin of IGM 13984. **(A)** Photography under UV light. **(B)** Schematic drawing. Abbreviations: Bpt, basipterygium; Fr, fin rays; Ma, medial arm; Mw, medial wing; Pp, posterior process; Ps, pelvic splint.

The pelvic fin is positioned opposite to the middle of the dorsal fin. The fin involves a conspicuous pelvic splint, a small ray, followed by a broad ray, then eight to 10 distally segmented and branched rays, which become smaller in lateromedial order (Fig 4B). The articular head of these rays is curved and sharp medially. The pelvic bones or basipterygium are triangular, about three times longer than wide, and are joined along their medial margin. In smaller specimens, the basipterygium consists only of a T-shaped keel, while in bigger specimens, this keel develops a broad lamellar medial wing. An indeterminate number of robust and short radials articulate to the posterior border of the pelvic bone and the head of the outermost pelvic rays.

#### Dorsal fin.

The dorsal fin is single and falcate. It extends above the centra 14 to 25, with variations. This fin consists of up to three small and unsegmented rays and 10-14 distally segmented and branched rays. Among these, the most anterior segmented ray is the longest and broadest; beyond, the posterior dorsal rays become progressively shorter.

**Fig 5:**
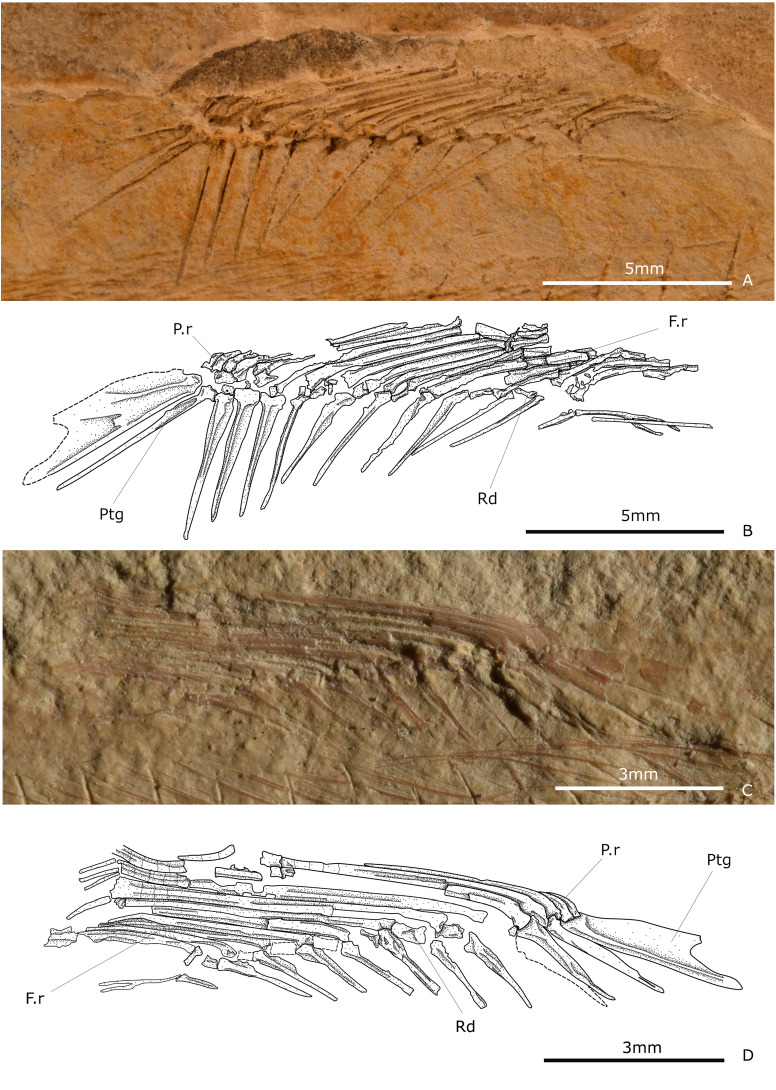
Dorsal fins. (A-B) Photography and schematic drawing of IGM 13987. (C-D) Photography and schematic drawing of IGM 13972. Abbreviations: Fr, fin rays; Pr, procurrent ray; Ptg, pterygiophore; Rd, radial.

The supporting pterygiophore series of this fin includes 11 to 13 long pterygiophores and small radials. In larger specimens, the first pterygiophore is large and has a complex shape with three thickened bars or arms anteroventrally projected (Fig 5B, D). The subsequent pterygiophores are rod-like bars that become smaller and thinner in anteroposterior order. The last one is a dorsal stay positioned horizontally.

#### Anal fin.

**Fig 6:**
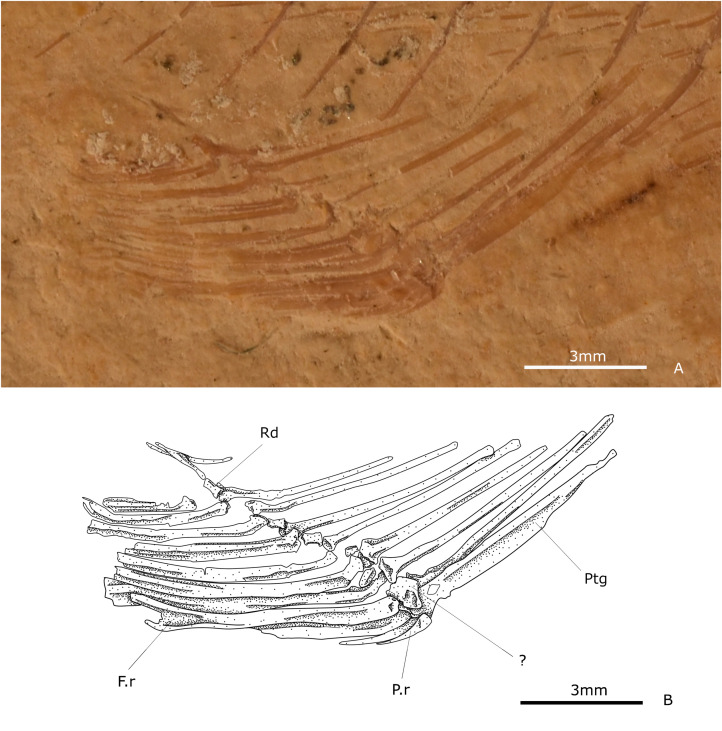
Anal fin. **(A)** Photography of IGM 13972. **(B)** Schematic drawing of IGM 13972. Abbreviations: Fr, fin rays; Pr, procurrent ray; Ptg, pterygiophore; Rd, radial.

The anal fin is single, falcate, and short (Fig 6A). It rises far in the back of the trunk, around the last fifth of the SL, below the centra 26 to 31, with variations. The fin consists of one to three rays and eight to 13 long, distally segmented, and branched rays (Fig 6A). Here, the second and third branched rays are the longest, and the others become progressively shorter. However, the length of the shortest is only slightly less than that of the longest.

The anal fin is supported by nine to 10 pterygiophores along small radials (Fig 6B). The pterygiophores are bar-like slender structures laterally flattened with small articular heads. In larger specimens, the anteriormost pterygiophore shows a broad laminar wing anteriorly expanded at the base of its length. In the anteroposterior direction, they exhibit a slight decrease in size. From the third pterygiophore, they penetrate each interhaemal space of the spines placed above. The last one is a bifurcated and horizontal anal stay. Radials are present between the articular heads of proximal pterygiophores. The first pterygiophore supports the procurrent rays and the first segmented ray.

#### Caudal skeleton.

**Fig 7:**
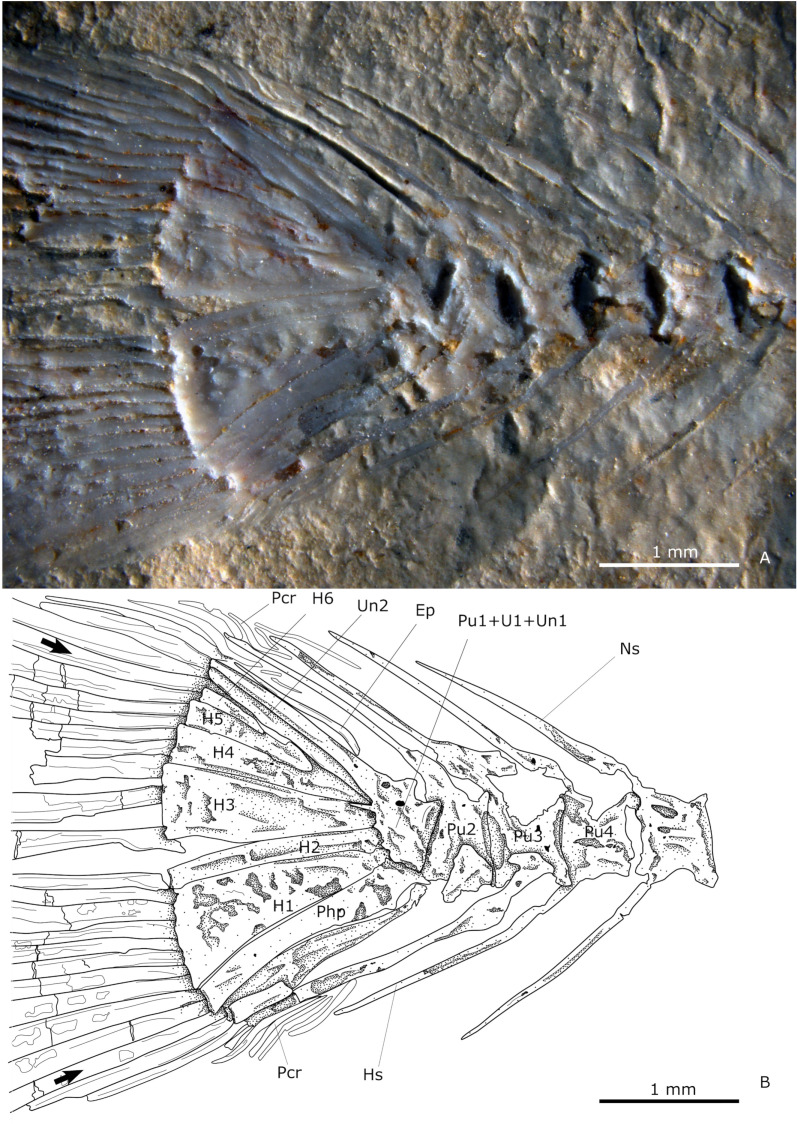
Caudal skeleton. **(A)** Photography of IGM 13984. **(B)** Schematic drawing of IGM 13984. Abbreviations: Ep, epurals; H1-6, hypural plates; Hs, haemal spine; Ns, neural spine; Pcr, procurrent ray; Php, procurrent ray; Pu1-4, preural centra; U, ural centra, Un1-2, uroneurals. Black arrows highlight the first main ray.

The caudal fin has two symmetrical, triangular, and deeply forked lobes. The posterior height of this fin exceeds the maximum height of the body. The caudal formula is ix + I + 8—8 + I + viii [64]. The parahypural and the haemal spine of preural 2 are broad and autogenous. Uroneural 1 is fused to preural centrum 1, and ural centra 1 and 2, forming a caudal complex or pleurostyle (Fig 7B). The uroneural 2 is located at the anterior edge of the pleurostyle, while an epural is situated at its posterior edge; both are elongated and autogenous (Fig 7B). Six autogenous hypurals decrease in length in ventrodorsal order, the first being the largest, triangular, and not fused (Fig 7B). The second one is significantly slenderer and rectangular. There is a diastema between the second and the third hypurals. From third to sixth, they are spatulate, slightly wider posteriorly.

#### Scales.

The trunk is entirely covered by cycloid, heart-shaped scales. These are slightly longer than high and have two similar anterior lobes. These scales have a central focus, numerous circuli ornamenting the anterior part of their lateral surfaces, and straight radii horizontally projected on the posterior half.

### Phylogenetic analysis

**Fig 8:**
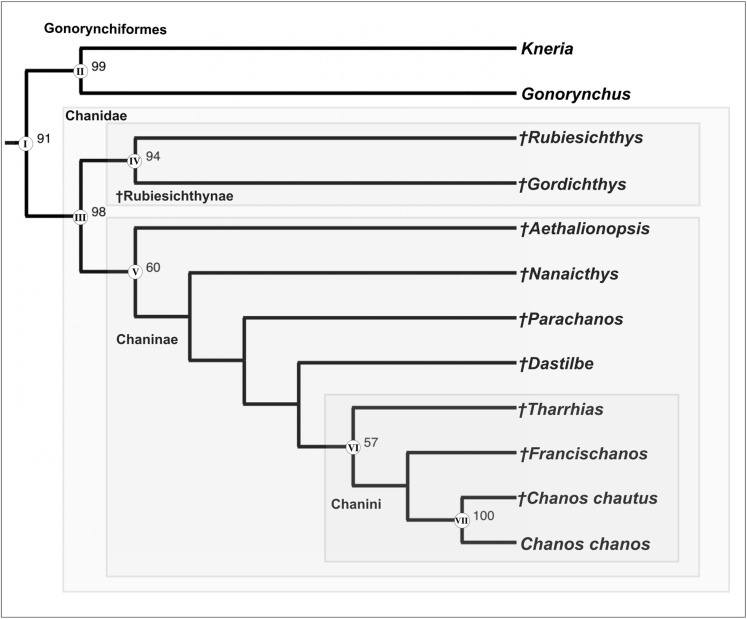
The single most parsimonious tree obtained in TNT v. **1.6**. Roman numbers on nodes indicate the main diagnostic clades mapped. Node Arabic numbers indicate > 50 bootstrap values.

A single most parsimonious tree (an ACCTRAN optimization, Fig 8) was recovered with a length of 170 steps (CI: 0.741; RI: 0.758). We describe the topology and character mapping of the main diagnostic clades Gonorynchiformes, Chanidae, Chaninae, Chanini, and *Chanos*.

Gonorynchiformes are monophyletic (node I) and supported in this hypothesis by seven characters: absence of orbitosphenoid (C: 1, 1); the presence of cephalic ribs articulating with the exoccipitals (C: 6, 1); reduced and flat blade-like parietals (C: 19, 1); absence of premaxillary ascending process (C: 28, 1); vomer extending anteriorly, beyond the level of the anterior margin of the mesethmoid (C: 45, 2); neural arch of first vertebrae in contact with exoccipital (C: 72, 1); and paired intermuscular bones consisting of three series (epipleurals, epicentrals, and epineurals) (C: 80, 1).

Gonorynchiformes is then divided into two clades, one that includes Kneriidae and Gonorynchidae (node II). Sister group to the aforementioned clade is equal to the taxonomic family Chanidae (node III), a monophyletic group supported by 11 characters: a large premaxilla, very broad, concave-convex, with a long oral process (C: 27, 1); enlarged posterior region of the maxilla, swollen to a bulbous outline with a curved posterior border (C: 33, 1); presence of a notch in the anterodorsal border of the dentary (C: 38, 1); quadrate-mandibular articulation anterior to orbit, quadrate displaced but not elongated (C: 48, 1); very long symplectic, about twice the length of the ingroup (C: 49, 1); presence of an anterior metapterygoid process of the hyomandibular bone (C: 52, 1); expanded opercular bone, at least one third of the head length (C: 54, 1); preopercular expansion distal to the terminal openings of the preopercular canal branches present and restricted to the posteroventral corner (C: 61, 1); first 5 to 10 neural arches autogenous in adults, at least laterally (C: 76, 1); extent of first uroneural to anterior end of preural centrum 2 (C: 95, 1); and autogenous haemal arch in preural centrum 2 (C: 103, 1).

Chanidae is then divided into two sister clades. The one grouping †*Rubiesichthys* +  †*Gordichthys* is equal to the subfamily †Rubiesychthynae (node IV), while the other corresponds to the taxonomic subfamily Chaninae (node V). The latter is supported by the presence of a maxillary process for articulation with the autopalatine (C: 32, 1); and a ridge present on the anteroventral limb of the preopercular bone (C: 60, 1).

Node VI is equal to the taxonomic tribe Chanini, whose members share three characters in this hypothesis: exoccipital bones with a posterior concave-convex border and a projection above the basioccipital (C: 5, 1); a large mesethmoid with broad posterolateral wing-like expansions (C: 10, 2), and the neural arch and spine of preural centrum 1 well developed, with the spine about half as long as preceding ones (C: 92, 0).

The last node (VII) is composed of the genus *Chanos*. The eight characters supporting this relationship are the presence of cephalic ribs articulating with both the exoccipitals and basioccipital (C: 6, 2); highly reduced parietals (C: 19, 2); the presence of caudal scutes (C: 90, 1); the fusion of ural centra (U1, U2), preural centrum 1 (Pu1), and uroneural 1 (Un1) in a pleurostyle or caudal complex (C: 91, 1); open neural arch and no spine in preural centrum 1 (C: 92, 2); uroneural 2 separated from ural centrum 2 (C: 96, 1); hypural 1 and terminal complex separated by a small hiatus (C: 100, 1), and the presence of a posterolateral process in the caudal endoskeleton (C: 104, 1). This hypothesis does not signal any autapomorphies for *C. chanos* or †*Chanos chautus* sp. nov.

### Geometric morphometrics

**Fig 9:**
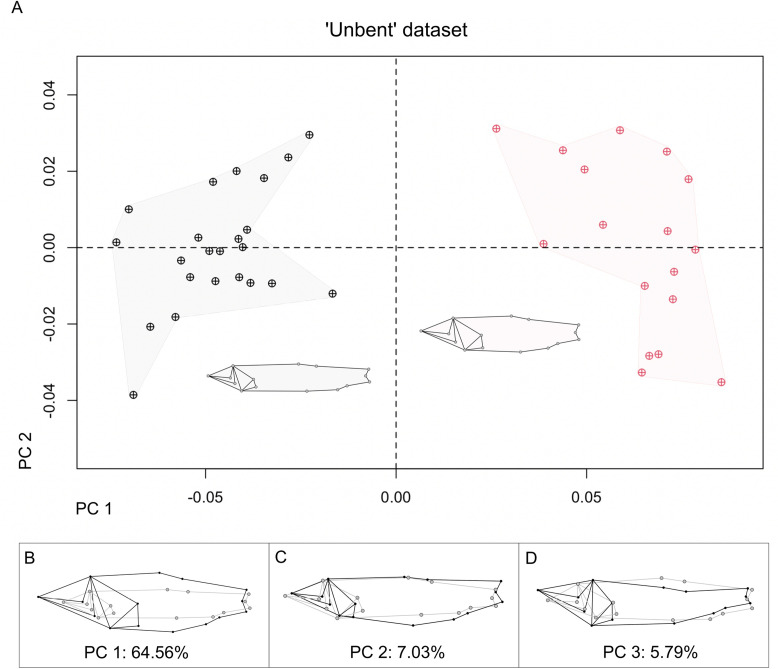
PCA on unbent dataset. **(A)** PC 1 vs PC 2 scatterplot. ‘M1’ specimens are in black, and ‘M2’ specimens are in light red, with the mean shape of the groups; clouds are delineated by eye. (B-**D)** PC 1-3 shape variations as differences between maximum (black) and minimum (grey), effects magnified x1.5 for visualization purposes.

We detected no outliers. The error was found not significant for either day (P = 0.5005) or specimens (P = 0.5005) (see Supporting information), indicating that the placement of landmarks was similar between replicates, and landmark estimation did not have any spurious effect. The first three principal components summarized 77.38% of shape variation on the unbent dataset. PC1 (64.56% of variance) describes relative head-size and body-depth ratios simultaneously. The PC1 vs PC2 scatter plot shows two groups: 23 specimens with more dorsoventrally compressed bodies and relatively smaller heads (morphotype 1) lie on negative values, while 17 specimens with deeper bodies and relatively bigger heads (morphotype 2) lie on the positive side of the cartesian plane (Fig 9A, B).

**Fig 10:**
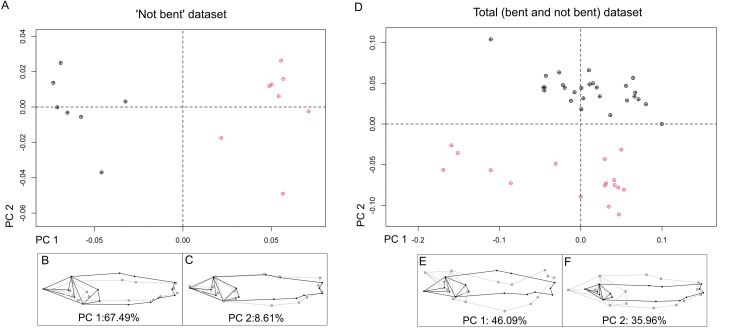
Exploratory PCAs. **(A)** Not bent dataset PC 1 vs PC 2 scatter plot. (B-**C)** Shape differences between maximum (black) and minimum (grey) for each PC. **(D)** ‘total’ dataset PC 1 vs PC 2 scatter plot. (E-**F)** Shape differences between maximum (black) and minimum (grey) for each PC. Effects magnified x1.2 for visualization purposes. M1 specimens in black and M2 specimens in light red.

PC2 (7.03% of variance) describes a subtle variation of anal-fin position, a higher positioned peduncle, and primarily related to pelvic fin depth, or a slightly more bulbous ‘belly’ for positive scoring specimens (Fig 9C). This variance does not seem as directly associated with arching as PC3, given that the exploratory PCA of the 15 not bent specimens also yielded a PC2 with the same pattern (Fig 10A–C). Nonetheless, it could still be related to preservation and or arching. PC3 (5.79% of variance) is correlated to residual upward bending on the positive scoring specimens (Fig 10D). In the exploratory PCA of the ‘total’ dataset, bending was the major axis of shape variation (PC1, 46.09%); meanwhile, the relative head size and body depth variation explained 35.96% of PC2 variance, still showing the morphotypes without overlap in PC1 vs PC2 scatter plot (Fig 10D–F).

**Fig 11:**
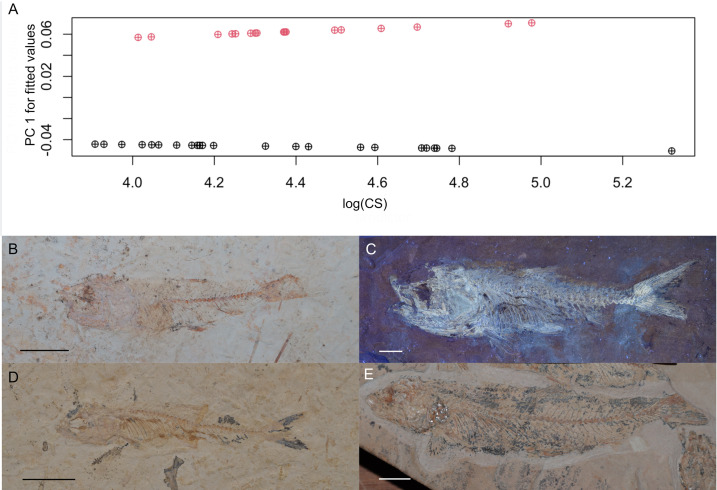
Allometric relationship between shape and size among morphotypes. **(A)** Allometric trajectories are visualized as the predicted lines of the first PC of shape and CS as the predictor. M2 specimens are in red, and M1 specimens are in black. **(B)** A small M2, IGM 14005. **(C)** A large M2, IGM 13978. **(D)** A small M1, IGM 14003. **(E)** A large M1, IGM 13974. Bars equal 10 mm.

For the ANOVA, differences between morphotype shapes were found significant (*P* < 0.05), while the effects of size and its interaction with morphotypes were found not significant (Table 2); the morphotypes showed similar size ranges. The restricted size ranges of the sample do not signal marked body-shape changes, as seen in the gentle slopes for the fitted shape values and the comparison between small and large-sized specimens in Fig 11.

**Table 2:** Results from exploratory permutational Procrustes ANOVA on morphotypes, size, and the interaction as factors.

	Df	SS	Rsq	F	*P*	*P* (99 permutations)
Morphotypes	1	0.11610	0.60127	56.9493	**0.001**	**0.01**
Size	1	0.00151	0.00782	0.7404	0.7	0.68
Morphotypes × size	1	0.00209	0.01083	1.0258	0.422	0.39
Residuals	36	0.00203	0.01083			
Total	39					

Significant effects are highlighted in boldface type. The last row shows *P* values from 99 permutations instead of 999.

**Fig 12:**
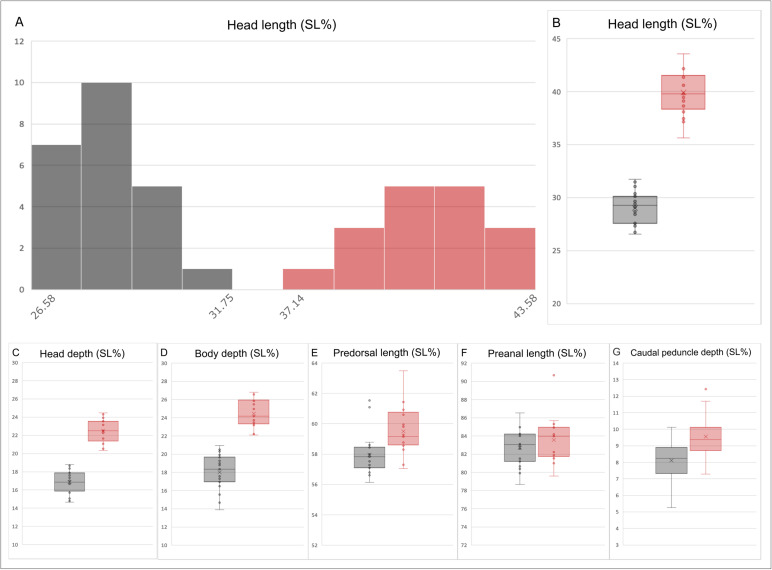
Landmark-based linear morphometrics graphics. **(A)** The head length frequency histogram of the total sample is expressed as SL%. (B-**G)** Mean deviations from within-group linear traits as SL%, error bar show 95% confidence intervals. M1 are in grey, while M2 are in light red.

Head length was the only landmark-based linear morphometric trait to show an unambiguous bimodal distribution in frequency histograms (Fig 12A). Box plots show that head length and depth and body depth show no distribution overlap (Fig 12B, C).

## Discussion

### †*Chanos chautus* sp. nov. systematic position

†*Chanos chautus* sp. nov. shows two of the morphological synapomorphies of Gonorynchiformes *sensu* Poyato-Ariza et al. [31]: the loss of the orbitosphenoid and the loss of the premaxillary ascending process. Other characters supporting the clade in our hypothesis include the presence of cephalic ribs, reduction of parietals, and paired intermuscular bones consisting of three series (epipleurals, epicentrals, and epineurals).

†*Chanos chautus* sp. nov*.* has several of the synapomorphies of Chanidae *sensu* Poyato-Ariza et al. [31]. These include a large, broad, concave-convex premaxilla with a spiny oral process; an enlarged maxilla, posteriorly bulbous and curved; the presence of a notch in the anterodorsal border of the dentary; a quadrate-mandibular articulation anteriorly displaced; a metapterygoid process in the hyomandibula, and an opercular bone expanded to at least one third the head length. Furthermore, the specimens possess other features that support the group in our hypothesis: a long symplectic (C: 49, 1); neural arches 5–10 autogenous, at least laterally (C: 76, 1); and haemal arch in preural centrum 2 autogenous (C: 103, 1).

†*Chanos chautus* sp. nov. (at least M1) has one synapomorphy of Chaninae *sensu* Poyato-Ariza et al. [31], a maxillary notch and process for articulation with the autopalatine (C: 32, 1), which also supports the group in our hypothesis (node V). They also possess a synapomorphy that characterizes the tribe Chanini *sensu* Poyato-Ariza et al. [31]: mesethmoid large, with broad posterolateral wing-like expansions; and the homoplastic characters: symplectic and quadrate separated through cartilage; preopercular expansion in the posteroventral corner and part of the posterodorsal limb. From our hypothesis (node VI), they also show exoccipitals with a posterior concave-convex border and a projection above basioccipital (C: 5, 1).

Regarding the genus *Chanos* (node VII), as defined in this hypothesis, †*Chanos chautus* sp. nov*.* shows cephalic ribs, but it is not clear where they articulate (C: 6, 2), caudal scutes are present in dorsal and ventral sections of the hypural plates and first rays at least in some specimens (e.g., IGM 13978, IGM 14022) (C: 90, 1); and hypural one and terminal centrum are at least not fused (C: 100, 1). Otherwise, †*Chanos chautus* sp. nov*.* unambiguously exhibit the remaining character states supporting the relationship: parietals are highly reduced (C: 19, 2); ural centra (U1, U2), preural centrum 1 (Pu1), and uroneural 1 (Un1) are fused (C: 91, 1); it has no spine in preural centrum 1, only the inferred pleurostyle (C: 92, 2); uroneural 2 is autogenous to the centrum (C: 96, 1); and possesses a posterolateral process in the caudal endoskeleton (C: 104, 1). Reduced parietals (C: 19, 2) and the posterolateral process of the caudal endoskeleton (C: 104, 1) are synapomorphic, but the rest of these characters also appear in *Kneria* Steindachner, 1866 and *Gonorynchus* (Linnaeus, 1766). Thus, they manifest as homoplasies in the analysis. Convergent evolution of most of the caudal osteology can ontologically explain this in extant genera [30]; consequently, the characters are congruent, and the affinity of †*Chanos chautus* sp. nov. with extant *C. chanos*, is unmistakable.

### †*Chanos chautus* sp. nov. and other fossil Chanidae

†*Caeus* Costa, 1857 from the Albian of Italy exhibits no fusion of caudal elements [12], and thus we unambiguously consider it a distinct taxon to †*Chanos chautus* sp. nov. Taverne and Capasso [12, p.13] suggested that †*Prochanos* Bassani, 1882 (Turonian-Maastrichtian, Croatia) might exhibit the fusion of caudal elements and be a *Chanos*, based on Bassani’s original illustration of the fossil [65, pl. 13]. However, the illustration of the caudal endoskeleton is not detailed. Moreover, Bassani [65, p. 218] erected a new genus based on four characters; one of them claims that the end of the vertebral column is more similar to that of †*Leptolepis* Agassiz, 1843 and †*Tharsis* Giebel, 1848 than to that of *Chanos*, likely referring to their not fused elements. The original description and diagnosis are general and superficial; therefore, the material needs re-description. Nonetheless, for the reasons outlined above, we consider †*Prochanos* tentatively distinct to *Chanos* (*sensu C. chanos* +  †*Chanos chautus* sp. nov.).

Most of the unarticulated elements of †*Vangus fahiny* Murray et al., 2023 (Mastrichthian, Madagascar) bear a striking resemblance to that of *Chanos.* Nevertheless*,* Murray et al. [16] considered the hyomandibula as a diagnostic feature of a new genus. The hyomandibula of †*Vangus* is higher and narrower, the head is angled less steeply and has a curved dorsal surface, with a rounder condyle for articulation with the opercle and a deeper concavity dorsal to the condyle, and a less rounded concave anterior side than that of *Chanos* [16, fig 2 A-D].

†*Cabindachanos dartevellei* Taverne, De Putter, Mees, Smith, 2019 was based on a single partial specimen missing the caudal skeleton from Cabinda (Democratic Republic of Congo) [15]. It is coeval (Danian or Salendian) to †*Chanos chautus* sp. nov. (Danian) [15]. The close relationship with *Chanos* is evident given the overall resemblance; for example, the kidney-like opercle and the well-developed and long supraoccipital crest are similar in shape yet hypertrophied. The parietals are reduced, as in *Chanos*, but are slightly wider than long. Furthermore, relative to the posterodorsal limb, the anteroventral limb of the preopercle is considerably narrower than in other chanids, such as †*Chanos chautus* sp. nov. The scales, as illustrated, also vary; they are longer than deep, with no clear focus [15, fig 5]. The loss of subopercle was cited as the major reason for erecting a new genus [15]; however, as implied by various specimens of †*Chanos chautus* sp. nov., this is most likely an artifact of preservation, as the subopercle is often covered by the opercle in our sample. Thus, although the level of affinity to the genus *Chanos* is difficult to attain, it is distinct to *C. chanos* or †*Chanos chautus* sp. nov.

Another Danian chanid found in literature is †*C. torosus* Danil’chenko, 1968 from Turkmenistan, but as for the Eocene and Oligocene of Italy, †*C. brevis*, †*C. zignoi* and †*C. forcipatus*, all need a modern description [2]. If these taxa were proven to be true *Chanos* species, the *Chanos* lineage ranged across the Tethys and the Proto-Caribbean earlier in the Cenozoic, at some point colonizing the Indo-Pacific Ocean before eventually going extinct in their former range.

### The morphotypes and *Chanos chanos*

A direct anatomical comparison of †*Chanos chautus* sp. nov. with extant *C. chanos* reveals a remarkable structural similarity of almost every visible element, suggesting that *Chanos* might have undergone morphological stasis in the last 63 million years. Bones such as, but not restricted to, the dentary, urohyal, intermusculars, cleithrum, supracleithrum, the entire caudal endoskeleton, supraneurals, pterygiophores, and the scales are indistinguishable from that of *C. chanos*. They exhibit an identical interfrontal depression and an elongated supraoccipital crest with a bifid set of filaments. The pterotics are long and have branch-like extensions. The fused autopalatine-ectopterygoid-endopterygoid complex is also seen in *C. chanos* e.g., [66, fig 10]. In all, the subopercle has an anterodorsal spiny process for articulation.

**Fig 13:**
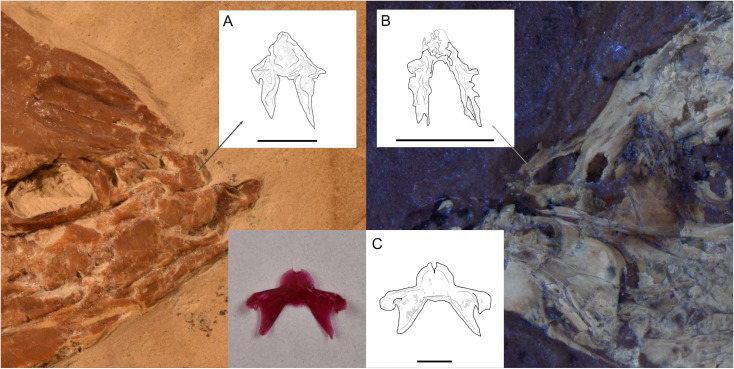
*Chanos* mesethmoids. **(A)** Schematic drawing and photography of IGM 13970 mesethmoid. **(B)** Schematic drawing and photography under UV light of IGM 13973 mesethmoid. **(C)** Schematic drawing and photography of *C. chanos* (CMR 1259 specimen) cleared and stained mesethmoid. All bars equal 3 mm.

Although it is only found in M1 specimens, we consider the mesethmoid shape as the only significant non-morphometric or meristic difference to distinguish between extant *Chanos* and †*Chanos chautus* sp. nov*.*, and therefore the only osteological qualitative difference to be included in the diagnosis. The anterior notch seen in the mesethmoid of extant *C. chanos* is missing in †*Chanos chautus* sp. nov. Also*,* the lateral wings in †*Chanos chautus* sp. nov are shorter and undeveloped, the posterior wings are longer, and their aperture is narrower (Fig 13).

**Fig 14:**
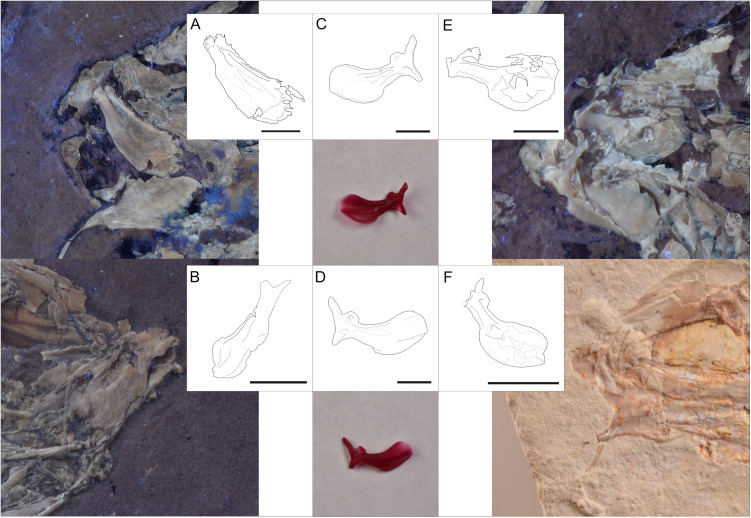
*Chanos* maxillae. **(A)** Schematic drawing and photography under UV light of IGM 13979 maxilla. **(B)** Schematic drawing and photography under UV light of IGM 13985 maxilla. (C-**D)** Schematic drawing and photography of *C. chanos* (CMR 1259 specimen) cleared and stained left maxilla in lingual and lateral view, respectively. **(E)** Schematic drawing and photography under UV light of IGM 13974 maxilla. **(F)** Schematic drawing and photography of IGM 14015 maxilla. All bars equal 2.5 mm.

Another consistent but mild difference is the upper jaw of M2, a straight bate-like maxilla (Fig 14A-B). The bulbous and curved posterior section of the maxilla of M1 resembles more closely that of *C. chanos* (Fig 14C-F). The shallow facet and process for autopalatine immediately behind the anterior processes seen in M1 and *C. chanos* is apparently missing in M2.

Grande and Arratia [30] reported a predorsal length for *C. chanos* of 51% of SL and a preanal length of 83%. Hence, with 56.14-63.49% of SL, there is a significant difference to those of †*Chanos chautus* sp. nov. for the first trait (Table 1). Preservation did not allow the use of meristics in multivariate analyses. However, selected meristic counts on some specimens generally fall within those of *C. chanos* extant populations, except for vertebral count (Table 3). There is no overlap between the vertebrae centra count of *C. chanos* (44-51) and †*Chanos chautus* sp. nov. (35-42), at least in the populations included. The number of centra slightly varies between morphotypes; there are 39 to 42 vertebrae, including the pleurostyle in M1 and 35 to 38 in M2. This centra variation is related to the dorsal fin position, extending from vertebra 18 to 25 in M1 and 14 to 21 in M2.

**Table 3:** Selected meristic counts.

	Vertebrae centra	Pectoral rays	Pelvic rays	Anal rays	Dorsal rays
†*Chanos chautus* sp. nov. (n = 18)	35-42	13-16	i, 9-11	i-iii, 8-13	i-iii, 10-14
†*Chanos chautus* sp. nov. M1 (n = 9)	38-42	15-16	i, 9-11	i-iii, 8-11	i-iii, 10-14
†*Chanos chautus* sp. nov. M2 (n = 9)	35-38	13-14	i,9	i-ii, 10-13	ii, 10-14
*C. chanos* Philipines	44-45	i, 13-17	i-ii, 7-12	i-iv, 6-9	ii-vi, 10-14
*C. chanos* Indonesia	44-45	16-17	11-12	10-11	14-16
*C. chanos* India	44	16	11	9-10	13-16
*C. chanos* Hawaii	45			ii, 9	ii, 12
*C. chanos* Papua New Guinea		16-17	11-12	9-11	13-17
*C. chanos* not specified	44-51	15-17	10-11	6-8	13-17

Extant *Chanos* data from [5,29]. Roman lowercase numbers refer to procurrent rays.

### Specimens as juveniles

*Chanos chanos* spawns in shallow near-shore waters across the tropical/subtropical Indo-Pacific [5]. The pelagic larvae (≈10mm) migrate to coastal transitional waters, e.g., estuaries or marshes that serve as nurseries [6]. Juvenile *C. chanos* feed on cyanobacteria, diatoms, algae, crustaceans, snails, worms, and zooplankton, seemingly preferring benthic items [5,6]. After a few months and a growth of around 300 mm of SL, the juveniles reach their habitat capacity and then return to the sea, where they add pelagic fish to their diet and can reach a length of up to one meter [5,6].

We infer that both fossil morphotypes represent juveniles since, in smaller specimens, all unpair fin pterygiophores and supraneurals are rod-like, while, in bigger specimens, the anterior pterygiophores and supraneurals show broad lamellar wings. Similarly, the pelvic bone in smaller specimens is a T-shaped bone, while in bigger specimens, the keel develops a lamellar medial wing. In addition to the shape changes, the general degree of ossification is also size-related. We attribute these size-related trends to skeletal maturation e.g., [67]. In combination with the size-related trends, we also consider the standard lengths of the sample, ranging from 35.8 to 147 mm, which are much smaller than those of mature extant milkfish [6,67].

Additionally, pending geological or geochemical evidence, we argue that the most parsimonious explanation is that the fossils were deposited in near-shore transitional waters. Our rationale follows: per our observations of the CNP material, only small-sized fish are found in both localities; the largest fossil fish specimen found in these localities is just 263.9 mm of SL, †*Kelemejtubus castroi* Cantalice and Alvarado-Ortega, 2017, a stem-percomorph [68]. Botanic material (plants and/or algae) is conspicuous and could have provided shelter for fish fry; however, these might not be necessarily autochthonous [22]. Most specimens are found well-preserved, semi- or articulated, semi- or complete, and in mass mortality events, indicating a low-energy and low-oxygen taphonomic scenario [69]. Some fishes reveal a strong marine influence, as some of the fossils come from generally reef-associated clades, such as the stem trumpetfish †*Eekaulostomus cuevasae* Cantalice and Alvarado-Ortega, 2016, the stem grouper †*Paleoserranus lakamhae* Cantalice, Alvarado-Ortega, Alaniz-Galvan, 2018, or the stem damselfish †*Chaychanus gonzalezorum* Cantalice, Alvarado-Ortega, Bellwood, 2020, as well as one undescribed taxon from the freshwater genus †*Phareodus* Leidy, 1873 [23,26,70,71]. Nonetheless, there are no fossil corals, and such fish taxa are rare, with only one or a handful of specimens. With dozens of juvenile specimens, by far the most common fossil fishes alongside chanids come from Anguilliformes and Clupeiformes, and several extant species of these groups are characterized by juveniles that migrate to transitional environments e.g., [72,73]. Given these characteristics, in this scenario, it is likely that at least at times and at least parts of Belisario Dominguez and División del Norte depositional environments lost their connection to the shallow sea, and oxygen depletion influenced the death and state of preservation of entrapped organisms e.g., [69,74]. Therefore, presumably juvenile †*Chanos chautus* sp. nov. also migrated to transitional environments, as does extant *C. chanos*.

### The *Chanos* morphotypes from Palenque

**Fig 15:**
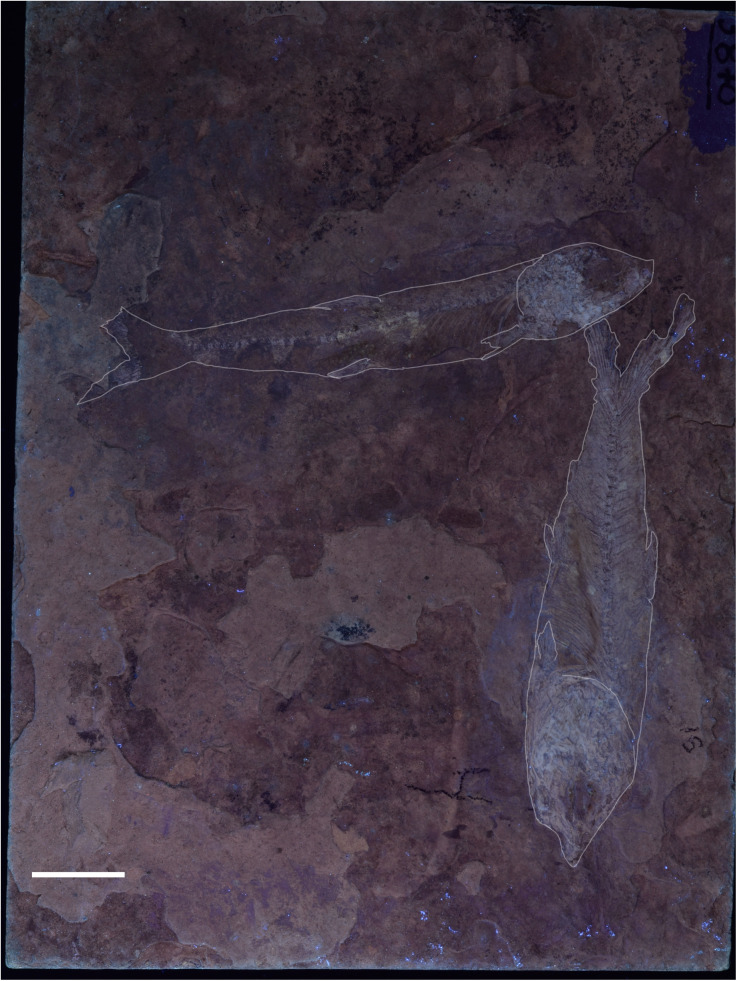
IGM 13977 (M1) and IGM 13984 (M2) association. Bar equals 10mm.

We discarded different scenarios that cannot account for the pattern of two morphotypes. Shape variation is not associated with a specific range of size, therefore, the differences in head size and body depth ratios are not products of allometric growth (Fig 11). Both morphotypes are found in both localities, hence, geographical variation of one taxon cannot account for them as suggested for †*Dastilbe* and other fossil species of *Chanos* [2,17–21] as well as for extant populations of *C. chanos* [7–10]. Despite not being usually conserved near one another, specimens IGM 13977 (M1) and IGM 13984 (M2) are close to one another on the same slab (Fig 15), so they must have coexisted in time and space. Consequently, other related scenarios, such as seasonal variation or anagenetic change, cannot explain morphotype variation.

Sexual shape dimorphism [63] could explain the presence of two morphotypes. It is a feature that has scarcely been discussed in fossil fish e.g., [22,69,75,76]. Using meristics and linear morphometrics, Poyato-Ariza [22] found two coexisting morphotypes of the chanid †*Rubiesichthtys gregalis*, which only differed in body depth. He linked the broader-bellied morphotype with extra space for ovaries in females, as seen in some extant fish e.g., [76,77]. We are unaware of any morphometric works concerning sexual shape dimorphism in extant *C. chanos*. Discrimination between sexes typically requires manipulation, although the abdomen may appear distended in gravid females [5]. In some populations, males tend to be smaller than females, while in others, there is no indication of sexual size dimorphism [5]. The sex ratio varies widely, yet males tend to be more common, 56-69% [5]. It is not clear if sexual maturity is related to age or size [5]. It varies across localities and types of stocks yet estimates show that sexual maturation takes years (3-10) and SL > 60 cm [5]. In *Chanos* morphotypes from Palenque, however, most of the shape variation is related to the head size, and only secondarily to body depth. Moreover, the maxilla presumably showed specific variation. The morphological traits perhaps associated with sexual dimorphism would have had to occur very early in development, and the trait would greatly diverge from the states seen in its close relatives *C. chanos*, †*Francischanos*, †*Tharrhias* or even †*Rubiesichthys* [14,22,78]. Nonetheless, slender M1 specimens are more common (57.5%), and sexual shape dimorphism can reflect other requirements that support reproductive success, such as food supply, locomotion, or mating behaviors [69].

A taxon characterized by polymorphism could also account for the morphotypes. For example, there are numerous resource polymorphisms in the teleost *Salvelinus alpinus* (Linnaeus, 1758) (Salmoniformes) [79]. These arctic char morphotypes show a wide range of genetic divergence; some, such as the Lake Hazen sympatric morphs, do not signal genetic differentiation [45]. It has been proposed that heritable foraging behaviors and plastic morphology might promote assortative mating and eventually lead to reproductive isolation in the arctic char [45,80]. Other *S. alpinus* morphs exhibit significant genetic differentiation, suggesting incipient speciation and the beginning of niche specialization [81].

The high morphometric differences and the slight osteological differences between morphotypes may signal early divergence or two well-established sympatric species (separately evolving metapopulation lineages, *sensu* DeQueiroz [82]). Chanids are morphologically conservative but characterized by high intraspecific and interspecific morphometric-meristic variability [14,17–21]. Given that the morphotypes represent juveniles, type differentiation could have been driven by some underlying genetic constitution that had a phenotypic effect since early ontogenetic stages and not only by ambient-induced plasticity. *Chanos chanos* is characterized by physiologic and phenotypic plasticity [5,10]. For example, abundance of food and current intensity were linked to differences in head, body, and caudal peduncle depths in juvenile milkfish along the coast of India [10]. However, these morphological stocks of *C. chanos* are not sympatric morphs but regional clusters [10,83]. Moreover, morphological stocks (regionally ambient-induced) do not coincide with molecular populations (related to genetic flow and geographic/current barriers) [8,10,83,84], which led Sri-Hari et al. [10,83] to suggest that morphological and genetic evolution are decoupled in the milkfish. Consequently, phenotypic differentiation may not necessarily signal genetic divergence in this case.

Our decision to erect a single new taxon instead of two was taxonomically pragmatic. †*Chanos chautus* sp. nov. may be a species complex, but we suggest a single species distinct to *C. chanos* as the null hypothesis for the presence of two *Chanos* morphotypes since any of the three scenarios, two species, a polymorphic, or a sexually dimorphic taxon is untestable with the available data. Hence, erecting a new taxon does not necessarily imply that †*Chanos chautus* sp. nov. was either a polymorphic or sexually dimorphic taxon. Despite the extreme osteological similarity between fossil and extant milkfishes, whether they were part of the direct ancestral lineage that led to the modern milkfish or whether they are *C. chanos* is also untestable; under a cladistic framework, fossil taxa are treated only as terminals and corroboration of the hypothesis would be based on the absence of characters [85,86].

### Head and depth variations

Both fossil morphotypes show terminal mouths and fusiform bodies overall, such as *C. chanos* and other generalist teleosts not associated with a particular zone of the water column [5,61]. However, it is still possible that they differed in some preference or performance related to their position in the water column. For example, teleosts characterized by polymorphism, such as *Perca fluviatilis* Linnaeus, 1758 (Perciformes) [87] and S. *alpinus* [88], show deeper bodies in benthic morphs and more streamlined bodies in pelagic morphs. Also, *Chanos* stocks of India with bigger heads, deeper bodies, and deeper caudal peduncles are probably the result of higher current intensity and high abundance of food [10].

Head and depth variation could also be linked to a differential foraging preference or performance [69,87,89], especially considering the morphotype-specific jaw variation. The elongated upper jaws and bigger heads in M2 could be adaptations for bigger food items or predatory adaptations for larger and slower prey when considering the bigger eyes and less streamlined shape in M2 [61]. A bigger head could also maximize buccal volume and suction velocity [90].

The distinct forms may have occupied different immediate ecological spaces due to resource competition [91,92]. Such spaces might have been available (e.g., emptied or rearranged) in the aftermath of the K/Pg mass extinction event, early into the radiation of the megadiverse Acanthomorpha e.g., [27,93]. Species may not occupy the entire niche space because of various exogenous and endogenous factors, including chance and interactions with other organisms [85]. This hypothesis remains consistent with the overall conservativism tendencies of *Chanos* seen in fry life history and osteological stasis. The fish was likely still generalist in other parameters, for example, euryhaline, as extant *C. chanos* and probably most of Chanidae [4,16]. Both morphotypes would still occupy the same fundamental niche, only varying or specializing [85]. *Chanos* may have an underlying genetic constraint that has prevented a higher osteological variation through protracted time. However, they can explore morphometric space through plasticity at least relatively more freely, and selection could have worked on the high-standing variation of those traits.

Moreover, these substrate or foraging scenarios are not only consistent with polymorphism or diverging lineages but also for sexual dimorphism, as differential niche occupancy driven by competition between sexes is seen in teleosts, such as *Jenynsia lineata* (Jenyns, 1842) (Cyprinodontiformes) [91]. Otherwise, such as *Decapterus macrosoma* Bleeker, 1851 (Perciformes), bigger heads and deeper bodies were associated with higher nutritional needs for egg production in females [94]. Alternatively, bigger heads and deeper bodies could reflect traits under sexual selection of competing males instead of being selected through resource utilization [78,94]. Another scenario that potentially explains the variation is perhaps different predation risks of adults [61,95].

## Conclusion

The findings represent the first fossil record of the depauperate Chanidae in North America, contributing to our knowledge of chanid diversity and disparity following the mass extinction event K/Pg. It expands the range of Paleocene *Chanos* from the eastern Tethys Ocean of today’s Turkmenistan to the western Proto-Caribbean of today’s Chiapas, Mexico. We successfully maximized the sample size by dealing with post-mortem body torsion and estimating missing landmarks, both representing useful tools for paleontological studies with usually imperfect specimens. †*Chanos chautus* sp. nov. is described as an association of two morphotypes. Given the osteological variation in the upper jaw and morphometric variation in head size and body depth, they may have differed in some foraging preferences. However, it is impossible to test whether the pattern can be better explained by either sexual dimorphism, polymorphism of one taxon, or two sympatric lineages. The *Chanos* morphotypes from Palenque were juvenile fish and pending other geological sources of evidence, we argue that the localities were, at least at times, deposited in transitional environments. Therefore, *Chanos* appears to have conserved life history traits alongside its overall morphology throughout most of the Cenozoic.

## Supporting information

S1 PDF Supplementary information. A pdf containing: a list of the referred material, including locality and the precise missing landmarks of each specimen from the GM sample. Table S1a. Landmark system; Fig S1a. Landmark system visualized; Table S1b. Linear measurements of each specimen from the unbent dataset; Fig S1b. The summary of the Error ANOVA. Character list.
(PDF)

S1 File Discrete morphological matrix. (NEX)

S2 File Raw total dataset. (TPS)

S3 File Digitization duplicate of the raw total dataset. (TPS)

S4 File Not-bent dataset. (TPS)

S5 File Unbent dataset. (TPS)

S6 File R code. (HTML)
