# Supplementary material for: Two contemporaneous morphs of fossil Chanos Lacepède, 1803 (Gonorynchiformes, Chanidae) from Paleocene (Danian) outcrops near Palenque (Mexico) revealed by geometric morphometrics indicate conservatism in milkfishes after the K/Pg boundary
Source: PLoS One. 2025 Mar 5;20(3):e0313912. doi: 10.1371/journal.pone.0313912 (PMC11882075; doi:10.1371/journal.pone.0313912)
Supplement: S1 PDF — Supplementary information. A pdf containing: a list of the referred material, including locality and the precise missing landmarks of each specimen from the GM sample. Table S1a. Landmark system; Fig S1a. Landmark system visualized; Table S1b. Linear measurements of each specimen from the unbent dataset; Fig S1b. The summary of the Error ANOVA. Character list. (PDF) [file pone.0313912.s001.pdf]

Supplementary information

Referred material

Housed in Colección Nacional de Paleontología, Instituto de Geología, Universidad Nacional Autónoma de México. Mexico City, Mexico. Accession numbers were created and updated in the manuscript after reviewing, in brackets are the reference tags used during our research for each specimen, all GM datasets still have a CH tag.

Total geometric morphometric sample.

- IGM 13970 (CH01). DN. 3 missing landmarks: 5, 6, 7.
- IGM 13971 (CH02). BD. 1 missing landmark: 14
- IGM 13972 (CH03). BD.
- IGM 13973 (CH04). DN. 4 missing landmarks: 5, 6, 7, 11.
- IGM 13974 (CH05). DN.
- IGM 13975 (CH06). DN. 1 missing landmark: 2.
- IGM 13976 (CH07). BD. 3 missing landmarks: 3, 4, 8.
- IGM 13977 (CH09). BD. 3 missing landmarks: 7, 8, 9.
- IGM 13978 (CH10). DN. 1 missing landmark: 1.
- IGM 13979 (CH11). BD. 1 missing landmark: 11.
- IGM 13980 (CH12). BD.
- IGM 13981 (CH13). BD.
- IGM 13982 (CH14). BD.
- IGM 13983 (CH15). BD.
- IGM 13984 (CH16). BD.
- IGM 13985 (CH17). DN. 2 missing landmarks: 8, 9.
- IGM 13986 (CH19). DN.
- IGM 13987 (CH20). DN.
- IGM 13988 (CH21). BD. 1 missing landmark: 10.
- IGM 13989 (CH22). BD.
- IGM 13990 (CH25). BD.
- IGM 13991 (CH26). BD. 1 missing landmark: 14.
- IGM 13992 (CH27). BD.
- IGM 13993 (CH28). BD. 1 missing landmark: 11
- IGM 13994 (CH29). DN. 1 missing landmark: 14
- IGM 13995 (CH30). BD.
- IGM 13996 (CH31). BD.
- IGM 13997 (CH32). BD.
- IGM 13998 (CH33). BD.
- IGM 13999 (CH34). BD.
- IGM 14000 (CH36). BD.
- IGM 14001 (CH37). BD.
- IGM 14002 (CH38). BD.
- IGM 14003 (CH39). BD.
- IGM 14004 (CH40). BD. 1 missing landmark: 2.
- IGM 14005 (CH41). BD.
- IGM 14006 (CH43). BD.

- IGM 14007 (CH44). BD.
- IGM 14008 (CH46). BD.
- IGM 14009 (CH47). BD.
- IGM 14010 (CH48). BD.
- IGM 14011 (CH50). DN. 1 missing landmark: 1.
- IGM 14012 (CH52). BD.
- IGM 14013 (CH53). BD.

Extra specimens used only as comparative material

- IGM 14014 (CH18). DN. An impression. Around 80 mm of SL. Lacks the dorsoposterior edge of the body outline.
- IGM 14015 (CH23). BD. Complete. Associated to a large juvenile eel. around 40 mm of SL.
- IGM 14016 (CH24). BD. Complete. Tail bent. Around 65 mm of SL. M1.
- IGM 14017 (CH35). DN. The largest specimen, around 180 mm of SL. The skull is heavily fragmented, missing the belly. By eye, a M1.
- IGM 14018 (CH42). BD. Complete. An impression, badly preserved. Around 80mm of SL. By eye, a M1.
- IGM 14019 (CH45). BD. Complete. A faint impression of 55 mm of SL.
- IGM 14020 (CH49). BD. Complete but distortion is highly sigmoidal, skull in dorsal view. Around 48 mm of SL.
- IGM 14021 (CH51). DN. By eye, a large M2, >150mm of SL. It is missing most of the head.
- IGM 14022 (CH54). DN. Only a well-preserved tail, maybe a M2.
- IGM 14023 (CH08). DN. Around 130 mm of SL. Big M2. Incomplete.

Table S1. Landmark system.

Num.	Type	Definition	Notes
1	ii	Snout tip	
2	ii	End of frontals	
3	i	Origin of dorsal fin	Positioned between first pterygiophore and first ray
4	i	Insertion (end) of dorsal fin	Positioned between last pterygiophore and last ray
5	ii	Origin of anteriormost dorsal procurrent caudal-fin ray	At the distal tip of the ray
6	i	End of the vertebral column	Between pleurostyle and hypural plate 1
7	ii	Origin of anteriormost ventral procurrent caudal-fin ray	At the distal tip of the ray
8	i	Insertion (end) of anal fin	Between last pterygiophore and last ray
9	i	Origin of anal fin	Between first pterygiophore and first ray
10	i	Origin of pelvic fin	Between basipterygium and first lateral ray

11	i	Origin of pectoral fin rays	Between basipterygium and first medial ray
12	i or ii	Cleithrum-supracleithrum articulation	In some specimens, it must be digitized as the posteriormost edge of the opercle
13	i or ii	Interopercle-subopercle articulation	In some specimens, it must be digitized as the anteroventralmost edge of opercle
14	ii	Angle between the arms of the preopercle's ridge	
15	ii	Ascending process of parasphenoid or posteroventral margin of the orbit	

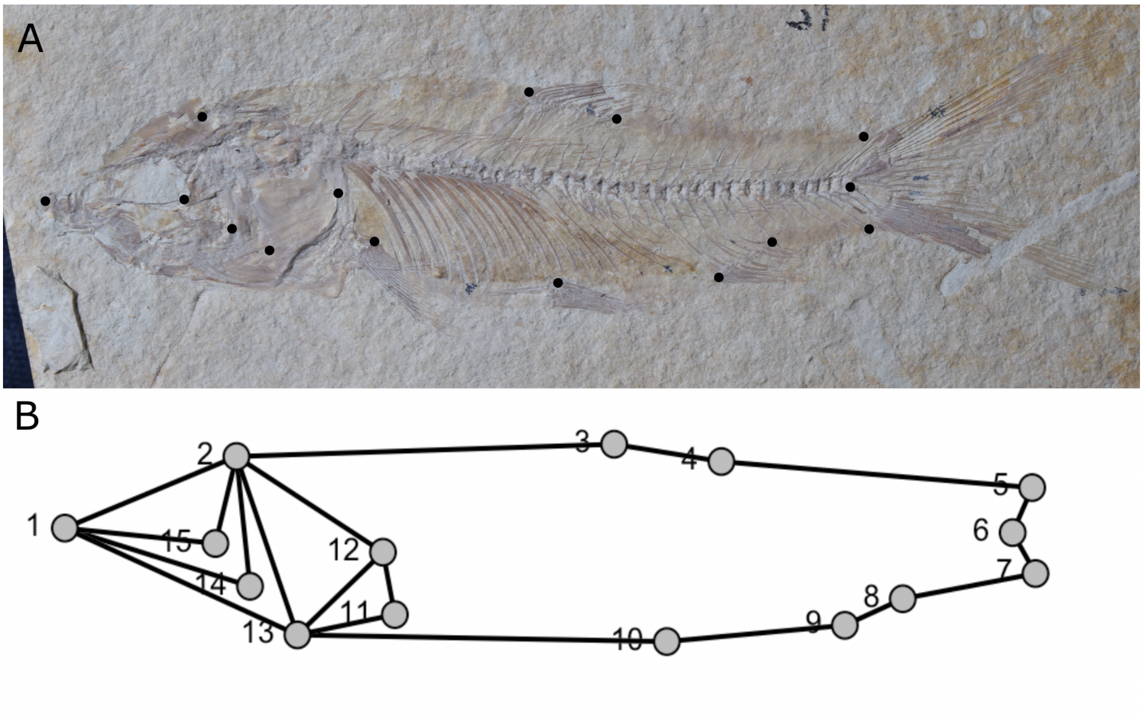

Fig S1. Landmark system visualized. A, landmarks digitized over CH12. B, landmark system over the mean shape of *Chanos chautus* sp nov.

Table S2. Linear measurements of each specimen from the unbent and estimated dataset.

	SL(mm)	HL(mm)	HL(SL%)	HD(mm)	HD(SL%)	BD(mm)	BD(SL%)	PD(mm)	PD(SL%)	PA(mm)	PA(SL%)	CPD(mm)	CPD(SL%)
CH01 (M1) DN	147.87	40.82	27.59	24.65	16.67	21.7	14.67	85.53	57.84	125.65	84.97	7.79	5.26
CH10 (M2) DN	115.22	47.91	41.58	27.56	23.91	25.58	22.2	68.91	59.8	94.76	82.24	11.24	9.75
CH43 (M2) BD	103.48	40.48	39.11	24.39	23.56	25.2	24.35	60.81	58.76	84.96	84.96	10.54	10.18
CH19 (M1) DN	88.36	26.22	29.67	14.89	16.85	15.35	17.37	50.62	57.28	69.53	78.68	7.29	8.25
CH06 (M1) DN	83.49	24.69	29.57	15.33	18.36	16.94	20.28	47.26	56.6	67.81	81.21	7.57	9.06
CH11 (M2) BD	83.44	33.88	40.6	19.32	23.15	22.05	26.05	48.94	58.65	70.88	84.94	10.4	12.42

CH20 (M1) DN	82.7	26.04	31.48	15.22	18.4	17.33	20.95	48.62	58.79	70.41	85.14	7.22	8.73
CH05 (M1) DN	82.32	23.58	28.64	14.38	17.46	15.65	19.01	48.2	58.55	68	82.6	5.4	6.55
CH04 (M1) DN	78.02	21.63	27.72	13.31	17.05	15.99	20.49	45.3	58	65.54	84	6.9	8.84
CH30 (M2) BD	72.82	25.94	35.62	15.08	20.7	19.34	26.55	42.45	58.29	61.14	83.96	8.45	11.6
CH50 (M2) DN	70.51	30.73	43.58	17.25	24.46	17.98	25.49	44.77	63.49	60.42	85.68	6.25	8.86
CH31 (M1) BD	69.66	18.52	26.58	10.21	14.65	12.8	18.37	40.71	58.44	58.56	84.06	5.53	7.93
CH25 (M2) BD	69.11	29.28	42.36	16.27	23.54	16.3	23.58	42.11	60.93	56.59	81.88	5.84	8.45
CH44 (M1) BD	67.98	19.78	29.09	10.34	15.21	13.36	19.65	39.75	58.47	58.82	86.52	5.23	7.69
CH21 (M1) BD	61.05	17.36	28.43	10.19	16.69	11.48	18.8	35.46	58.1	48.79	79.91	4.48	7.33
CH53 (M2) BD	60.77	25.21	41.48	12.82	21.09	14.31	23.54	37.34	61.44	55.11	90.68	5.69	9.36
CH12 (M2) BD	59.5	22.1	37.14	12.09	20.32	14.86	24.97	36.32	61.04	50.09	84.18	6.96	11.69
CH40 (M2) BD	59.48	22.66	38.09	14.46	24.31	15.88	26.69	33.94	57.06	48.19	81.01	5.57	9.36
CH29 (M1) DN	58.14	16	27.51	8.76	15.06	11.83	20.34	32.64	56.14	46.9	80.66	5.34	9.18
CH13 (M2) BD	56.59	22.53	39.81	12.6	22.26	15.16	26.79	33.15	58.57	46.2	81.66	5.46	9.64
CH27 (M2) BD	56.56	23.39	41.35	12.63	22.33	13.67	24.17	33.18	58.66	46.54	82.28	5.13	9.07
CH04 (M1) BD	55.39	20.76	37.47	11.99	21.64	12.99	23.45	32.84	59.28	44.09	79.59	4.34	7.83
CH09 (M1) BD	55.03	16.34	29.69	9.54	17.33	8.08	14.68	33.86	61.53	45.74	83.11	3.94	7.15
CH16 (M2) BD	53.99	22.77	42.17	11.71	21.68	11.93	22.09	32.37	59.95	44.31	82.07	5.44	10.07
CH41 (M2) BD	53.64	20.73	38.64	11.01	20.52	12.73	23.73	30.74	57.3	43.74	81.54	4.66	8.68
CH17 (M2) DN	50.81	20.64	40.62	11.85	23.32	11.8	23.22	30.76	60.61	43.35	85.31	3.7	7.28
CH33 (M1) BD	48.26	14.14	29.29	7.66	15.87	8.22	17.03	27.42	56.81	39.88	82.63	3.64	7.54
CH52 (M1) BD	46.71	13.74	29.41	7.79	16.67	7.87	16.49	26.92	57.63	38.88	83.23	3.91	8.37
CH38 (M1) BD	46.23	14.05	30.39	8.64	18.68	8.88	19.21	26.83	58.03	39	84.36	4.46	9.64
CH46 (M1) BD	46.15	12.61	27.32	7.33	15.88	7.88	17.07	28.19	61.08	38.45	83.31	3.81	8.255
CH02 (M1) BD	45.96	12.61	27.43	7.22	15.7	6.39	13.9	26.45	57.55	36.94	80.37	3.8	8.26
CH03 (M1) BD	44.53	14.14	31.75	7.84	17.6	8.87	19.91	25.23	56.65	37.53	84.28	4.51	10.12

CH15 (M2) BD	43.3	17.09	39.46	10.08	23.27	11.2	25.86	25.4	58.66	36.51	84.31	4.12	9.51
CH39 (M1) BD	42.53	13.21	31.06	7.16	16.83	6.62	15.56	24.93	58.61	34.73	81.66	2.99	7.03
CH22 (M2) BD	42.11	16.55	39.3	9.48	22.51	9.77	23.2	24.91	59.15	35.45	84.18	3.68	8.73
CH26 (M1) BD	41.83	12.6	30.12	6.19	14.79	7.1	16.97	24.24	57.94	33.43	79.91	3.4	8.12
CH37 (M1) BD	40.42	12.23	30.25	7.6	18.8	7.8	19.29	23.17	57.32	33.51	82.9	3.6	8.9
CH47 (M1) BD	38.61	11.08	28.69	6.81	17.68	6.77	17.56	22.21	57.52	32.07	83.06	2.78	7.2
CH34 (M1) BD	36.8	9.84	26.73	6.59	17.9	6.37	17.31	20.9	56.79	30	81.52	2.92	7.93
CH48 (M1) BD	35.89	10.56	29.42	6.45	17.97	7.06	19.67	20.49	57.09	30.23	84.22	3.35	9.33
Mean			33.655		19.266		20.762		58.61		83.069		8.728

SL, standard length (interlandmark distance 1-6); HL, head length (interlandmark distance 1-12); HD, head depth (interlandmark distance 2-13); BD, body depth (interlandmark distance 3-10); PD, predorsal length (interlandmark distance 1-3); PA, preanal length (interlandmark distance 1-9); CPD, caudal peduncle depth (interlandmark distance 5-7).

Error

```
Analysis of Variance, using Residual Randomization
Permutation procedure: Randomization of null model residuals
Number of permutations: 1000
Estimation method: Ordinary Least Squares
Sums of Squares and Cross-products: Type I
Effect sizes (Z) based on F distributions

                Df       SS      MS     Rsq  F  Z Pr(>F)
Specimens       28 72031307 2572547 0.99856  0  0 0.5005
Days             1     7450    7450 0.00010  0  0 0.5005
Specimens:Days  28    96356    3441 0.00134  0  0 0.5005
Residuals        0        0    -Inf 0.00000
Total           57 72135113

Call: procD.lm(f1 = coords ~ Specimens * Days, iter = 999, data = DfrError1)
```

FigS2. Screenshot of the ANOVA's summary.

Character list, from Ribeiro et. al. (2018).

Cranium

1. Orbitosphenoid: present [0], absent [1].
2. Basisphenoid: present [0], absent [1].
3. Pterosphenoids: well developed and articulating with each other [0], slightly reduced, not articulating anteroventrally but approaching each other anterodorsally [1], greatly reduced and broadly separated both anteroventrally and anterodorsally [2].
4. Posterolateral expansion of exoccipitals: absent [0], present [1].
5. Exoccipitals: posteriorly smooth with no projection above the basioccipital [0], with a posterior concave-convex border, and a projection above basioccipital [1].
6. Cephalic ribs: absent [0], present and all articulating with the exoccipitals [1], present and articulating with both the exoccipitals and basioccipital [2].
7. Supraoccipital crest: small, short in lateral view [0]; long and enlarged, projecting above occipital region and first vertebrae, forming a vertical, posteriorly deeply pectinated blade [1].
8. Foramen magnum: dorsally bounded by exoccipitals [0]; enlarged and dorsally bounded by supraoccipital [1].
9. Brush-like cranial intermuscular bones (*sensu* Patterson, Johnson, 1995): absent [0], present [1].
10. Mesethmoid: wide and short [0]; long and slender, with anterior elongate lateral extensions [1]; large, with broad posterolateral wing-like expansions [2].
11. Wings (extensions) of lateral ethmoids: absent [0]; present [1].
12. Nasal bone: small but flat [0]; just a tubular ossification around the canal [1].
13. Frontals: wide through most of their length, narrowing anteriorly to form a triangular anterior border [0]; elongate and narrow except in postorbital region [1]; wide, anteriorly shortened, anterior border roughly straight [2].
14. Interfrontal fontanelle: absent [0]; present [1].
15. Frontal bones: paired in adult [0]; co-ossified, with no median suture [1].
16. Foramen for olfactory nerve in frontal bones: absent [0]; present [1].
17. Relative position of the parietals: medioparietal (in full contact with each other along their midline) [0]; mesoparietal (*sensu* Poyato-Ariza, 1994); partly separated by the supraoccipital, posteriorly, and partly in contact with each other, anteriorly) [1]; lateroparietal (completely separated from each other by the supraoccipital) [2].
18. Parietal portion of the supraorbital canal: absent [0]; present [1].
19. Parietals: large [0]; reduced but flat and blade-like in shape [1]; highly reduced [2]; absent as independent ossifications [3].

Orbital region

20. Number of infraorbitals: five or more [0]; four [1]; three or fewer [2].
21. Infraorbital bones not including lacrimal: well developed [0]; reduced to small, tubular ossifications [1].
22. Lacrimal: flat and comparable in length to subsequent infraorbitals [0]; flat, long and large, with keel near lower edge [1].
23. Supraorbital: present [0]; absent [1].

Jaws

24. Teeth in premaxilla, maxilla, and dentary: present [0]; absent [1].
25. Premaxilla: consisting of one solid element [0]; premaxilla consisting of two distinct elements, with a shorter, non-osseous element lying ventral to a much longer osseous portion, which in turn articulates with the maxilla [1].
26. Premaxillary "gingival teeth": absent [0]; present [1].
27. Premaxilla: small, flat and roughly triangular [0]; large, very broad, concave-convex, with long oral process [1]; narrow and elongated, its length more than one half of the length of the maxilla [2].
28. Premaxillary ascending process: present [0]; absent [1].
29. Morphology of maxillary articular process: thin and pointed [0]; robust and bulky [1]; flat and hypertrophied, higher than the main body of the bone [2].
30. Length of maxillary articular process: short, less than 30% of the total maxillary length [0]; long, 30%-40% of the total maxillary length [1]; very long, about 50% of the total maxillary length [2].
31. Dorsal and ventral borders of the maxillary articular process: straight or slightly curved [0]; very curved, almost describing an angle [1].
32. Maxillary process for articulation with autopalatine: absent [0]; present [1].
33. Posterior region of the maxilla: slightly and progressively expanded to form a thin blade, with roughly straight posterior border [0]; very enlarged, swollen to a bulbous outline, with curved posterior border [1].
34. Supramaxilla(e): present [0]; absent [1].
35. Symphysis: low, pointed [0]; higher than immediately posterior part of the dentary, robust [1].
36. Notch between the dentary and the anguloarticular bones: absent [0]; present [1].
37. Articulation between dentary and angulo-articular: strong, dentary not V-shaped posteriorly [0]; loose, with a posteriorly V- shaped dentary [1].
38. Notch in the anterodorsal border of the dentary ("leptolepid" notch): absent [0]; present [1].
39. Mandibular sensory canal: present [0]; absent [1].

Palate & suspensorium

40. Dermopalatine: present [0], absent [1].
41. A patch of about 20 conical teeth on endopterygoid and basibranchial 2: absent [0]; present [1].
42. Ectopterygoid: well developed, ectopterygoid overlapping with the ventral surface of the autopalatine by at least 50% [0]; reduced, articulating with the ventral surface of the autopalatine by at most 10% through cartilage, resulting in a loosely articulated suspensorium [1].
43. Teeth on vomer and parasphenoid: absent [0]; present [1].
44. Anterior portion of vomer: horizontal [0]; anteroventrally inclined, nearly vertical [1]; dorsally curved [2].
45. Spatial relationship between vomer and mesethmoid anteriorly: vomer and mesethmoid ending at about the same anterior level [0]; mesethmoid extending anteriorly beyond the level of anterior margin of vomer [1]; vomer extending anteriorly beyond the level of anterior margin of mesethmoid [2].
46. Metapterygoid: large, broad and in contact with quadrate and symplectic through cartilage [0]; reduced to a thin rod [1].
47. Quadrate: with posterior margin smooth [0]; elongated forked posterior process [1].

48. Quadrate-mandibular articulation: below or posterior to orbit, no elongation or displacement of quadrate [0]; anterior to orbit, quadrate displaced but not elongate [1].
49. Symplectic: elongated in shape but relatively short [0]; very long, about twice the length of the ingroup [1].
50. Symplectic and quadrate: articulating directly with each other [0]; separated through cartilage [1].
51. Articular head of hyomandibular bone: double, with both articular surfaces placed on the dorsal border of the main body of the bone [0]; double, with the anterior articular surface forming a separate head from the posterior articular surface [1].
52. Metapterygoid process of hyomandibular bone: absent [0], present, anterior [1]; present, ventral [2].
53. Ossified interhyal: present [0]; absent as an independent ossification [1].

Opercular series

54. Size of opercular bone: normal, about one quarter of the head length [0]; expanded, at least one third of the head length [1].
55. Shape of opercular bone in lateral view: rounded/oval [0]; triangular [1].
56. Opercular apparatus on external surface of opercle: absent [0]; present [1].
57. Opercular borders: free from side of head [0]; partly or almost completely connected to side of head with skin [1].
58. Angle formed by preopercular limbs: obtuse [0]; approximately straight [1]; acute [2].
59. Posterodorsal limb of preopercular bone: well developed [0]; reduced, correlated with expansion of anteroventral limb that meets its fellow along the ventral midline [1].
60. Ridge on anteroventral limb of preopercular bone: absent [0]; present [1].
61. Preopercular expansion distal to the terminal openings of the preopercular canal branches: absent, preopercular bone not enlarged [0]; present, restricted to the posteroventral corner [1]; present in posteroventral corner and part of the posterodorsal limb [2].
62. Suprapreopercular bone: absent [0]; present as a relatively large, flat bone [1]; present as tubular ossification(s) [2].
63. Major axis of subopercular bone in lateral view: inclined [0]; subhorizontal [1].
64. Subopercular clefts: absent [0]; present [1].
65. Posterodorsal ascending process of interopercular bone: absent [0]; present [1].

Branchial arches

66. Teeth on fifth ceratobranchial: present [0]; absent [1].
67. First basibranchial in adult specimens: ossified [0]; unossified [1].
68. Fifth basibranchial in adult specimens: cartilaginous [0]; ossified [1].
69. First pharyngobranchial in adult specimens: ossified [0]; unossified [1].

Vertebrae

70. Two anteriormost vertebrae: as long as posterior ones [0]; shorter than posterior ones [1].
71. Autogenous neural arch anterior to first vertebra: present [0]; absent [1].
72. Neural arch of first vertebra and exoccipitals: separate [0]; in contact [1].
73. Neural arch of first vertebra and supraoccipital: separate [0]; in contact [1].
74. Spine on neural arch of first vertebra: present, well developed [0]; present but reduced [1].
75. Anterior neural arches: no contact with adjoining arches [0]; abutting contact laterally with adjoining arches, no overlapping [1].

76. Neural arches 5–10 in adults: fused to centra [0]; autogenous, at least laterally [1].
77. Neural arches to vertebrae posterior to the dorsal fin in adults: fused to centrum [0]; autogenous, at least laterally [1].
78. First two anterior parapophyses: autogenous [0]; fused to centra [1].
79. Rib on third vertebral centrum: similar in size and shape to posterior ones [0]; widened and shortened [1]; modified into Weberian apparatus [2].

Intermuscular bones

80. Paired intermuscular bones consisting of three series: epipleurals, epicentrals, and epineurals: absent (at least one complete series is absent) [0]; present (three series) [1].
81. Anterior (first six) epicentral bones: unmodified, no differences in size from others [0]; highly modified, much larger than posterior ones [1].
82. Shape of anterior supraneurals 1 and 2: narrow and separated [0]; large and in contact [1].
83. Posterior process on the posterior border of first supraneural: absent [0]; present [1].
84. Number of supraneurals: several supraneurals in a long series [0]; two or fewer supraneurals [1].

Girdles and fins

85. Postcleithra: present [0]; absent [1].
86. Lateral line and supracleithrum: supracleithrum pierced through dorsal region [0]; supracleithrum pierced all through its length [1]; lateral line does not pierce supracleithrum [2].
87. Fleshy lobe of paired fins: absent [0]; present [1].
88. Caudal fin morphology: elongated, posteriorly forked [0]; higher than long, slightly incurved posteriorly [1].
89. Fringing fulcra in dorsal lobe of caudal fin: present [0]; absent [1].
90. Caudal scutes: absent [0]; present [1].

Caudal endoskeleton

91. Ural centra (u1, u2), preural centrum one (pu1), and uroneural one (un1): autogenous [0]; fused [1].
92. Neural arch and spine of preural centrum one: both well developed, spine about half as long as preceding ones [0]; arch complete and closed, spine rudimentary [1]; arch open, no spine [2].
93. Uroneurals (regardless of number): arranged in a linear series [0]; arranged in a double series [1].
94. Total number of uroneurals regardless of their fusion to other elements of the caudal endoskeleton (dealt with in character 92 above): three [0]; two [1]; one [2].
95. Anterior extent of first uroneural: to anterior end of first preural [0]; to anterior end of second preural [1]; to anterior end of third preural [2]; uroneural fused to caudal fin complex [3].
96. Uroneural two and second ural centrum: in contact [0]; separated [1]; uroneural two absent as an autogenous ossification [2].
97. Parhypural and preural centrum 1: independent in adults [0]; fused only in large adults [1]; fused since early ontogenetic stages [2].
98. Reduction in the number of hypurals: six [0]; fewer than six [1].
99. Hypurals 1 and 2: autogenous [0]; partly fused to each other [1].
100. Hypural 1 and terminal centrum: articulating [0]; separated by a hiatus [1]; fused [2].
101. Hypural 2 and centrum: fused [0]; autogenous [1].

102. Hypural 5 and second ural centrum: separate [0]; articulating [1].
103. Hemal arch in preural centrum 2: fused to the centrum [0]; autogenous [1].
104. Posterolateral process of caudal endoskeleton: absent [0]; present [1].

Scales and lateral line

105. Type of scales: cycloid [0]; modified ctenoid [1].
106. Lateral line: not extending to posterior margin of hypurals [0]; extending to posterior margin of hypurals [1].
