# Supplementary material for: Two contemporaneous morphs of fossil Chanos Lacepède, 1803 (Gonorynchiformes, Chanidae) from Paleocene (Danian) outcrops near Palenque (Mexico) revealed by geometric morphometrics indicate conservatism in milkfishes after the K/Pg boundary
Source: PLoS One. 2025 Mar 5;20(3):e0313912. doi: 10.1371/journal.pone.0313912 (PMC11882075; doi:10.1371/journal.pone.0313912)
Supplement: S6 File — (HTML) [file pone.0313912.s007.html]

Chanos chautus GM


# Chanos chautus GM

#### Guadarrama-Pérez, Alberto

#### 2024-05-23

```
## 
## Attaching package: 'dplyr'
```

```
## The following objects are masked from 'package:stats':
## 
##     filter, lag
```

```
## The following objects are masked from 'package:base':
## 
##     intersect, setdiff, setequal, union
```

```
## Loading required package: RRPP
```

```
## Loading required package: rgl
```

```
## Loading required package: Matrix
```

Error

```
setwd("/Users/alberto/Desktop/ParaTPS/PaperScript/FINAL/2024")

D1<-readland.tps("totalsample.TPS", specID = "ID", negNA = TRUE)
```

```
## 
## No curves detected; all points appear to be fixed landmarks.
## 
## Warning: not all specimens have scale adjustment (perhaps because they are already scaled);
## no rescaling will be performed in these cases
```

```
D1est<-estimate.missing(D1, method = c("TPS", "Reg"))
D1proc<-gpagen(D1est, print.progress = F)

D2<-readland.tps("totalsampleb.TPS", specID = "ID", negNA = TRUE)
```

```
## 
## No curves detected; all points appear to be fixed landmarks.
## 
## Warning: not all specimens have scale adjustment (perhaps because they are already scaled);
## no rescaling will be performed in these cases
```

```
D2est<-estimate.missing(D2, method = c("TPS", "Reg"))


D1D2<-abind(D1, D2)
D1D2est<-abind(D1est,D2est)


rep(c("Day1", "Day2"), times= c(44,44)) %>% as.factor() -> Days

paste0("specimen", 1:44) %>% rep(2) %>% as.factor() -> Specimens 


DfrError1<-geomorph.data.frame(coords=D1D2, Days= Days, Specimens= Specimens)
DfrError2<-geomorph.data.frame(coords=D1D2est, Days= Days, Specimens= Specimens)


ANOVAraw<-procD.lm(f1= coords ~ Specimens * Days, data = DfrError1, iter = 999)
ANOVArawB<-procD.lm(f1= coords ~ Specimens * Days, data = DfrError1, iter = 99)

summary(ANOVAraw)
```

```
## 
## Analysis of Variance, using Residual Randomization
## Permutation procedure: Randomization of null model residuals 
## Number of permutations: 1000 
## Estimation method: Ordinary Least Squares 
## Sums of Squares and Cross-products: Type I 
## Effect sizes (Z) based on F distributions
## 
##                Df       SS      MS     Rsq  F  Z Pr(>F)
## Specimens      28 72031307 2572547 0.99856  0  0 0.5005
## Days            1     7450    7450 0.00010  0  0 0.5005
## Specimens:Days 28    96356    3441 0.00134  0  0 0.5005
## Residuals       0        0    -Inf 0.00000             
## Total          57 72135113                             
## 
## Call: procD.lm(f1 = coords ~ Specimens * Days, iter = 999, data = DfrError1)
```

```
summary(ANOVArawB)
```

```
## 
## Analysis of Variance, using Residual Randomization
## Permutation procedure: Randomization of null model residuals 
## Number of permutations: 100 
## Estimation method: Ordinary Least Squares 
## Sums of Squares and Cross-products: Type I 
## Effect sizes (Z) based on F distributions
## 
##                Df       SS      MS     Rsq  F  Z Pr(>F)
## Specimens      28 72031307 2572547 0.99856  0  0  0.505
## Days            1     7450    7450 0.00010  0  0  0.505
## Specimens:Days 28    96356    3441 0.00134  0  0  0.505
## Residuals       0        0    -Inf 0.00000             
## Total          57 72135113                             
## 
## Call: procD.lm(f1 = coords ~ Specimens * Days, iter = 99, data = DfrError1)
```

```
ANOVAestimated<-procD.lm(f1= coords ~ Specimens * Days, data = DfrError2, iter = 99)
ANOVAestimatedB<-procD.lm(f1= coords ~ Specimens * Days, data = DfrError2, iter = 99)


summary(ANOVAestimated)
```

```
## 
## Analysis of Variance, using Residual Randomization
## Permutation procedure: Randomization of null model residuals 
## Number of permutations: 100 
## Estimation method: Ordinary Least Squares 
## Sums of Squares and Cross-products: Type I 
## Effect sizes (Z) based on F distributions
## 
##                Df        SS      MS     Rsq  F  Z Pr(>F)
## Specimens      43 118164349 2748008 0.99850  0  0  0.505
## Days            1      8192    8192 0.00007  0  0  0.505
## Specimens:Days 43    168818    3926 0.00143  0  0  0.505
## Residuals       0         0    -Inf 0.00000             
## Total          87 118341358                             
## 
## Call: procD.lm(f1 = coords ~ Specimens * Days, iter = 99, data = DfrError2)
```

```
summary(ANOVAestimatedB)
```

```
## 
## Analysis of Variance, using Residual Randomization
## Permutation procedure: Randomization of null model residuals 
## Number of permutations: 100 
## Estimation method: Ordinary Least Squares 
## Sums of Squares and Cross-products: Type I 
## Effect sizes (Z) based on F distributions
## 
##                Df        SS      MS     Rsq  F  Z Pr(>F)
## Specimens      43 118164349 2748008 0.99850  0  0  0.505
## Days            1      8192    8192 0.00007  0  0  0.505
## Specimens:Days 43    168818    3926 0.00143  0  0  0.505
## Residuals       0         0    -Inf 0.00000             
## Total          87 118341358                             
## 
## Call: procD.lm(f1 = coords ~ Specimens * Days, iter = 99, data = DfrError2)
```

exploratory not bend PCA

```
setwd("/Users/alberto/Desktop/ParaTPS/PaperScript/FINAL/2024")

notbent<-readland.tps("notBent.TPS", specID = "ID", negNA = TRUE)
```

```
## 
## No curves detected; all points appear to be fixed landmarks.
```

```
notbentproc<-gpagen(notbent, print.progress = F)

plotOutliers(notbentproc$coords)
```

```
## CH50  CH09  CH34  CH11  CH33  CH04  CH20  CH16  CH53  CH27  CH17  CH48  CH43  
##    14     3     5     8     4     2     7    10    15    12    11     6    13 
## CH03  CH12  
##     1     9
```

```
PCAa<-gm.prcomp(notbentproc$coords)

rep(c("M1", "M2"), times= c(7,8)) %>% as.factor() -> Cola

plotPCAa<-plot(PCAa, col=Cola, pch=10)
```

```
Linkscsv<-read.csv("links.csv", header = FALSE)
LinksChanos15<-as.matrix(Linkscsv)

plotRefToTarget(PCAa$shapes$shapes.comp1$min,
                PCAa$shapes$shapes.comp1$max,
                method = "points",
                mag = 1,
                links = LinksChanos15,
                axes = F,
                label = F)
```

```
plotRefToTarget(PCAa$shapes$shapes.comp2$min,
                PCAa$shapes$shapes.comp2$max,
                method = "points",
                mag = 1,
                links = LinksChanos15,
                axes = F,
                label = F)
```

exploratory total PCA

```
setwd("/Users/alberto/Desktop/ParaTPS/PaperScript/FINAL/2024")


PCAb<-gm.prcomp(D1proc$coords)

rep(c("M1", "M2"), times= c(27,17)) %>% as.factor() -> Colb

plotPCAb<-plot(PCAb, col=Colb, pch=10)
```

```
Linkscsv<-read.csv("links.csv", header = FALSE)
LinksChanos15<-as.matrix(Linkscsv)

plotRefToTarget(PCAb$shapes$shapes.comp1$min,
                PCAb$shapes$shapes.comp1$max,
                method = "points",
                mag = 1,
                links = LinksChanos15,
                axes = F,
                label = F)
```

```
plotRefToTarget(PCAb$shapes$shapes.comp2$min,
                PCAb$shapes$shapes.comp2$max,
                method = "points",
                mag = 1,
                links = LinksChanos15,
                axes = F,
                label = F)
```

Main analysis

```
setwd("/Users/alberto/Desktop/ParaTPS/PaperScript/FINAL/2024")

Unbent<-readland.tps("totalunbent.TPS", specID = "ID", negNA = TRUE)
```

```
## 
## No curves detected; all points appear to be fixed landmarks.
```

```
Unbentproc<-gpagen(Unbent, print.progress = F)

plotOutliers(Unbentproc$coords)
```

```
## CH50  CH10  CH11  CH01  CH25  CH02  CH40  CH16  CH27  CH53  CH17  CH31  CH13  
##    39    24    25     1    33     2    36    30    34    40    31    13    27 
## CH46  CH15  CH43  CH41  CH09  CH44  CH14  CH34  CH22  CH30  CH29  CH04  CH33  
##    21    29    38    37     7    20    28    15    32    35    12     4    14 
## CH05  CH19  CH12  CH20  CH47  CH26  CH21  CH37  CH52  CH03  CH38  CH39  CH06  
##     5     8    26     9    22    11    10    17    23     3    18    19     6 
## CH48  
##    16
```

```
PCAc<-gm.prcomp(Unbentproc$coords)

rep(c("M1", "M2"), times= c(23,17)) %>% as.factor() -> Colc

PCAplot<-plot(PCAc, col=Colc, pch=10)
```

```
plot(PCAc, col=Colc, axis1 = 1, axis2 = 3, pch=10)
```

```
Linkscsv<-read.csv("links.csv", header = FALSE)
LinksChanos15<-as.matrix(Linkscsv)

plotRefToTarget(PCAc$shapes$shapes.comp1$min,
                PCAc$shapes$shapes.comp1$max,
                method = "points",
                mag = 1.5,
                links = LinksChanos15,
                label = F)
```

```
plotRefToTarget(PCAc$shapes$shapes.comp2$min,
                PCAc$shapes$shapes.comp2$max,
                method = "points",
                mag = 1.5,
                links = LinksChanos15,
                label = F)
```

```
plotRefToTarget(PCAc$shapes$shapes.comp3$min,
                PCAc$shapes$shapes.comp3$max,
                method = "points",
                mag = 1.5,
                links = LinksChanos15,
                label = F)
```

```
Meanshape<-mshape(Unbentproc$coords)
msM1<-mshape(Unbentproc$coords[,,1:23])
msM2<-mshape(Unbentproc$coords[,,23:40])


plotRefToTarget(Meanshape,
                Meanshape,
                method = "points",
                label = "T",
                links = LinksChanos15)
```

```
plotRefToTarget(msM1,
                msM2,
                method = "TPS",
                 mag = 1.5,
                links = LinksChanos15)
```

```
plotRefToTarget(msM1,
                msM1,
                method = "points",
                links = LinksChanos15)
```

```
plotRefToTarget(msM2,
                msM2,
                method = "points",
                links = LinksChanos15)
```

Exploratory ANOVA

```
UnbentDfr<-geomorph.data.frame(Shape= Unbentproc$coords, Morphotypes= Colc, Size= Unbentproc$Csize)

ANOVAunbent<-procD.lm(f1= Shape ~ Morphotypes*log(Size), iter=999, data = UnbentDfr, print.progress = F)
ANOVAunbentB<-procD.lm(f1= Shape ~ Morphotypes*log(Size), iter=99, data = UnbentDfr, print.progress = F)

summary(ANOVAunbent)
```

```
## 
## Analysis of Variance, using Residual Randomization
## Permutation procedure: Randomization of null model residuals 
## Number of permutations: 1000 
## Estimation method: Ordinary Least Squares 
## Sums of Squares and Cross-products: Type I 
## Effect sizes (Z) based on F distributions
## 
##                       Df       SS       MS     Rsq       F       Z Pr(>F)   
## Morphotypes            1 0.116107 0.116107 0.60127 56.9493  4.1384  0.001 **
## log(Size)              1 0.001509 0.001509 0.00782  0.7404 -0.5209  0.700   
## Morphotypes:log(Size)  1 0.002091 0.002091 0.01083  1.0258  0.2225  0.422   
## Residuals             36 0.073396 0.002039 0.38009                          
## Total                 39 0.193104                                           
## ---
## Signif. codes:  0 '***' 0.001 '**' 0.01 '*' 0.05 '.' 0.1 ' ' 1
## 
## Call: procD.lm(f1 = Shape ~ Morphotypes * log(Size), iter = 999, data = UnbentDfr,  
##     print.progress = F)
```

```
summary(ANOVAunbentB)
```

```
## 
## Analysis of Variance, using Residual Randomization
## Permutation procedure: Randomization of null model residuals 
## Number of permutations: 100 
## Estimation method: Ordinary Least Squares 
## Sums of Squares and Cross-products: Type I 
## Effect sizes (Z) based on F distributions
## 
##                       Df       SS       MS     Rsq       F       Z Pr(>F)  
## Morphotypes            1 0.116107 0.116107 0.60127 56.9493  4.3756   0.01 *
## log(Size)              1 0.001509 0.001509 0.00782  0.7404 -0.4702   0.68  
## Morphotypes:log(Size)  1 0.002091 0.002091 0.01083  1.0258  0.2930   0.39  
## Residuals             36 0.073396 0.002039 0.38009                         
## Total                 39 0.193104                                          
## ---
## Signif. codes:  0 '***' 0.001 '**' 0.01 '*' 0.05 '.' 0.1 ' ' 1
## 
## Call: procD.lm(f1 = Shape ~ Morphotypes * log(Size), iter = 99, data = UnbentDfr,  
##     print.progress = F)
```

```
plotAllometry(ANOVAunbent, size =Unbentproc$Csize, logsz = T, method = "PredLine", col= Colc, pch=10)
```

```
plotAllometry(ANOVAunbent, size =Unbentproc$Csize, logsz = T, method = "RegScore", col= Colc, pch=10)
```

```
plotAllometry(ANOVAunbent, 
              size =Unbentproc$Csize, 
              logsz = T, 
              method = "size.shape", 
              col= Colc, 
              pch=10)
```

```
plotAllometry(ANOVAunbent, 
              size =Unbentproc$Csize, 
              logsz = T, 
              method = "CAC", 
              col= Colc, 
              pch=10)
```

```
#Calculating shapes of either end of allometric trend for each morphotype

#M1
DfrM1Allo<-geomorph.data.frame(ShapeM1=Unbentproc$coords[,,1:23], Size=Unbentproc$Csize[1:23])

AllometryM1<-procD.lm(f1= ShapeM1~log(Size), iter=999, data = DfrM1Allo, print.progress = F)

AllometryPlotM1<-plot(AllometryM1,
                    type = "regression",
                    predictor = log(DfrM1Allo$Size),
                    reg.type = "RegScore")
```

```
predictionsM1<-shape.predictor(AllometryM1$GM$fitted,
                             x=AllometryPlotM1$RegScore, Intercept = F,
                             PredMin= min(AllometryPlotM1$RegScore),
                             PredMax= max(AllometryPlotM1$RegScore))

#M1 visualization
plotRefToTarget(predictionsM1$PredMin, predictionsM1$PredMax, method = "vector", mag = 3, links = LinksChanos15)
```

```
#M2
DfrM2Allo<-geomorph.data.frame(ShapeM2=Unbentproc$coords[,,24:40], Size=Unbentproc$Csize[24:40])

AllometryM2<-procD.lm(f1= ShapeM2~log(Size), iter=999, data = DfrM2Allo, print.progress = F)

AllometryPlotM2<-plot(AllometryM2,
                    type = "regression",
                    predictor = log(DfrM2Allo$Size),
                    reg.type = "RegScore")
```

```
predictionsM2<-shape.predictor(AllometryM2$GM$fitted,
                             x=AllometryPlotM2$RegScore, Intercept = F,
                             PredMin= min(AllometryPlotM2$RegScore),
                             PredMax= max(AllometryPlotM2$RegScore))


#M2 visualization
plotRefToTarget(predictionsM2$PredMin, predictionsM2$PredMax, method = "vector", mag = 3, links = LinksChanos15)
```
